# TODO: A Triple‐Outcome Double‐Criterion Optimal Design for Dose Monitoring‐and‐Optimization in Multi‐Dose Randomized Trials

**DOI:** 10.1002/sim.70090

**Published:** 2025-05-19

**Authors:** Jingyi Zhang, Heng Zhou, Nolan A. Wages, Zifang Guo, Fang Liu, Thomas Jemielita, Fangrong Yan, Ruitao Lin

**Affiliations:** ^1^ Research Center of Biostatistics and Computational Pharmacy China Pharmaceutical University Nanjing China; ^2^ Biostatistics and Research Decision Sciences Merck & Co., Inc Rahway New Jersey USA; ^3^ Department of Biostatistics Virginia Commonwealth University Richmond Virginia USA; ^4^ Department of Biostatistics The University of Texas MD Anderson Cancer Center Houston Texas USA

**Keywords:** cohort expansion, dose monitoring, dose optimization, dynamic linear model, noninferiority, triple‐outcome decision

## Abstract

Detecting the efficacy signal and determining the optimal dose are critical steps to increase the probability of success and expedite the drug development in cancer treatment. After identifying a safe dose range through phase I studies, conducting a multidose randomized trial becomes an effective approach to achieve this objective. However, there have been limited formal statistical designs for such multidose trials, and dose selection in practice is often ad hoc, relying on descriptive statistics. We propose a Bayesian optimal two‐stage design to facilitate rigorous dose monitoring and optimization. Utilizing a flexible Bayesian dynamic linear model for the dose–response relationship, we employ dual criteria to assess dose admissibility and desirability. Additionally, we introduce a triple‐outcome trial decision procedure to consider dose selection beyond clinical factors. Under the proposed model and decision rules, we develop a systematic calibration algorithm to determine the sample size and Bayesian posterior probability cutoffs to optimize specific design operating characteristics. Furthermore, we demonstrate how to concurrently assess toxicity and efficacy within the proposed framework using a utility‐based risk‐benefit trade‐off. To validate the effectiveness of our design, we conduct extensive simulation studies across a variety of scenarios, demonstrating its robust operating characteristics.

## 1 Introduction

In oncology, early‐phase trials have traditionally followed the “toxicity‐then‐efficacy” paradigm, wherein sponsors tend to select a higher dose within an acceptable safety range for subsequent clinical studies with the belief that it may lead to better treatment outcomes. This approach stems from the scarcity of cancer patients and the urgent unmet clinical needs in oncology. However, such decisions regarding dosage can potentially have negative impacts. One major concern is the relatively short follow‐up period in early‐phase studies, which hinders a comprehensive evaluation of the drug's safety and tolerability. This is especially problematic for targeted therapies that require multiple cycles of administration, as the long‐term toxicity is often overlooked in early dose‐finding trials. Consequently, further registrational studies or real‐world data may reveal significant safety or tolerability issues associated with unnecessarily high doses, necessitating backfilling studies to re‐evaluate the dosage. In response to safety concerns highlighted by real‐world data on drug usage, drugs with multiple mechanisms of action have undergone dose modifications or additions even after obtaining U.S. Food and Drug Administration (FDA) approval. Examples include Ceritinib (a small‐molecule drug), Cabazitaxel (chemotherapy), and Gemtuzumab ozogamicin (an antibody‐drug conjugate) [1]. For patients, receiving a high dose may increase the probability of experiencing adverse events, leading to a diminished quality of life or treatment discontinuation. Moreover, lower doses may be nearly as efficacious as higher doses. Therefore, dose optimization plays a crucial role in maximizing the benefits for patients. Recognizing the significance of dose optimization and its insufficiency in practice, regulatory agencies have taken proactive steps to address this issue. To this end, the FDA has issued the *Project Optimus* initiative, emphasizing the importance of evaluating the dose–response relationship through a multidose randomized trial and determining the optimal dose through a comprehensive risk‐benefit assessment [2].

While the crucial role of conducting multidose randomized trials for dose optimization is widely recognized, discussions on rigorous statistical methods for such trials are quite limited. Iasonos and O'Quigley [3] proposed a model‐based approach to assess both the toxicity and efficacy of a dose expansion cohort and determine recommended phase II doses in a frequentist framework. Mokdad et al. [4] proposed monitoring toxicity and efficacy for dose‐expanded cohorts using confidence bounds, which are predefined in an ad hoc way. Wang and Tan [5] developed a multistage dose‐expansion cohort that combines frequentist sample size determination with Bayesian safety monitoring. The dose selection in the methods mentioned above is determined by comparing toxicity or efficacy rates to fixed threshold values without including between‐dose comparisons. This is mainly due to the fact that between‐dose comparisons typically necessitate a larger sample size to achieve desirable statistical power.

Our research is motivated by the dose escalation [6, 7] and dose selection [8] trials of Belantamab mafodotin for relapsed or refractory multiple myeloma. Belantamab mafodotin is an antibody‐drug conjugate containing a B‐cell maturation antigen‐targeting antibody. In the dose escalation phase, DREAMM‐1, a total of ten doses (ranging from 0.03 to 4.6 mg/kg IV every 3 weeks) were investigated, and 38 patients were recruited, yet the maximum tolerated dose was not reached. Afterward, a two‐dose randomized comparative trial (DREAMM‐2) was conducted to identify the optimal dose for further evaluation. In this study, a total of 196 patients were randomized to one of the two doses (2.5 and 3.4 mg/kg IV every 3 weeks), with the objective response rate as the primary endpoint. A Bayesian futility monitoring procedure was used to eliminate futile dose arms. If both arms passed the futility monitoring, a Bayesian dose‐comparison posterior probability was calculated to identify the superior dose. Notably, the decision boundaries for futility monitoring and dose‐comparison posterior probability were not systematically optimized in this study. Moreover, conducting a large‐scale trial with 196 patients in the exploratory phase may be impractical for many pharmaceutical companies due to resource constraints. Existing designs, especially those developed for large‐sample randomized studies like the one used in the DREAMM2 study, may be less effective in trials with moderate sample sizes. Therefore, our goal is to develop a flexible design that accommodates a feasible and reasonable sample size while still yielding meaningful results for most multidose randomized trials.

In addition to the strategy of borrowing information across doses through the construction of a dose–response model, the existing literature has investigated several approaches to effectively reduce the sample size required for a randomized comparative study. Sargent et al. [9] and Hong and Wang [10] proposed using three trial outcomes, including an inconclusive region, as opposed to the conventional binary decisions of rejecting or not rejecting the null hypothesis. This approach reduces the sample size by relaxing the stringent requirements of frequentist error rates. The three‐outcome rule has been used in several other phase II clinical trial designs. For example, Frewer et al. [11], Roychoudhury et al. [12], and Zhao et al. [13] have proposed designs that incorporate three outcomes by evaluating both statistical significance and clinical relevance. Another strategy to reduce sample size is to use a dual‐criterion approach that combines the standard between‐arm comparison with a one‐sample rejection decision rule for two‐sample phase II trials [14]. The dual‐criterion approach results in a more stringent rule for rejecting the null hypothesis under unpromising or marginally promising efficacy rates but a less stringent rule for promising rates. As a result, the dual‐criterion approach is capable of controlling the type I error rate while simultaneously achieving high statistical power, even with a small sample size. However, never before has a method for multidose randomized trials combined the three aforementioned strategies, especially under the Bayesian framework.

To fill this gap, we first propose to employ the Bayesian dynamic linear model (DLM) to exploit information from the dose–response relationship. The DLM approach has been applied in various research fields, including economics, industry, science, and socio‐economics, and has shown promising potential in non‐oncology dose ranging trials [15, 16, 17, 18]. We utilize a generalized framework to explore the dose–response relationship to deal with multidose studies in oncology, leveraging the DLM's ability to associate adjacent doses and its flexibility in accommodating nonmonotone dose–response relationships.

Finding the optimal dose in multidose trials is challenging due to the potential plateau effect of the dose–efficacy curve. To address this, we employ dual criteria: The first criterion corresponds to per‐dose futility/toxicity monitoring, requiring that admissible doses demonstrate superiority over historical controls or the standard of care in terms of efficacy or safety. The second criterion involves between‐dose noninferiority comparisons. More specifically, we define the optimal dose as the lowest dose that offers comparable efficacy to the highest admissible dose. The underlying principle is to select a lower dose if the loss in efficacy is acceptable in anticipation of achieving a more favorable risk‐benefit tradeoff. The concept shares similarities with noninferiority (NI) trials, which assess whether a new treatment is not unacceptably less efficacious than a reference control treatment. A prespecified NI margin is established to reflect the amount of tolerable difference in efficacy. Demonstrating the NI is appropriate when the experimental treatment is not expected to be superior in efficacy to current treatments but may offer advantages such as lower cost, ease of administration, or a better safety profile. In our scenario, the optimal dose is the lowest dose that is noninferior to the highest dose in terms of efficacy, with the potential advantage of reducing the incidence of adverse events for patients. Existing studies on NI trials offer a range of methods to meet various clinical requirements [19, 20, 21, 22, 23, 24, 25, 26]. The majority of these methods are designed for confirmatory trials within the frequentist framework and require an unrealistically large sample size for early‐phase trials. Since formal hypothesis testing is not suitable for exploratory trials due to limited sample sizes, we adopt the concept of the NI test and develop a Bayesian approach for comparing doses. Moreover, we utilize a triple‐outcome decision rule [9, 10, 27, 28] to enable clinicians to incorporate other clinical considerations into the decision‐making procedure when evidence of the NI is insufficient. The triple‐outcome decision rule allows for a more comprehensive assessment, accounting for additional clinical considerations beyond efficacy or toxicity comparisons.

The remainder of this article is organized as follows: In Section 2, we introduce the dynamic linear model and the trial design. In Section 3, we introduce the triple‐outcome double‐criterion design. In Section 4, we provide a systematic calibration method for parameter optimization. Section 5 presents comprehensive simulation studies to evaluate the performance of the proposed design, compare it with alternative approaches, examine the influence of design parameters, and conduct sensitivity analysis to assess its robustness. In Section 6, we extend the proposed method to jointly evaluate toxicity and efficacy using a utility function. Section 7 provides a brief discussion.

## 2 Bayesian Dynamic Linear Model

Consider a multidose randomized trial with J candidate doses of a treatment, denoted as $d_{1}<\cdots <d_{J}$, carefully selected based on prior phase I trials or other relevant information. In practice, the choice of J depends on considerations such as trial cost, duration, and patient recruitment challenges. The study doses should not exceed the maximum tolerated dose established in prior phase I trials. Therefore, we assume that all study doses have an acceptable toxicity profile, with dose selection primarily driven by efficacy outcomes. However, it is worth noting that incorporating toxicity data into the proposed design is straightforward, as demonstrated in Section 6.

In practice, most early‐phase oncology studies use a binary efficacy endpoint (e.g., the objective response) denoted as Y to assess the short‐ or intermediate‐term efficacy of the cancer treatment. Let $p_{j}=\mathrm{Pr}(Y=1|d_{j})$ be the true efficacy rate of dose $d_{j}$. We employ the dynamic linear model, a curve‐free (or nonparametric) approach particularly suitable for scenarios with only a few candidate doses, to quantify the dose–efficacy relationship. Let $\left(y_{j},n_{j}\right)$ represent the observed data at dose j, where $y_{j}$ is the number of efficacy outcomes, and $n_{j}$ is the number of evaluable patients. The Bayesian dynamic linear model is expressed as

(1) $$\begin{array}{ll} y_{j}\;|\;p_{j} & ∼Bin(n_{j},p_{j}),\;j=1,\ldots ,J\\ \mu_{j} & =\Phi^{-1}(p_{j}),\;j=1,\ldots ,J\\ \mu_{1} & ∼N(\theta ,\sigma_{1}^{2})\\ \mu_{j}\;|\;\mu_{j-1},\sigma_{j} & ∼N(\mu_{j-1},\sigma_{j}^{2}),\;j=2,\ldots ,J\\ \sigma_{j} & ∼HC(\xi ,\tau^{2}),\;j=2,\ldots ,J \end{array}$$

In the above dynamic linear model, $\Phi^{-1}(p_{j})$ is the probit transformation, based on the inverse of the cumulative distribution function of the standard normal random variable, that transforms $p_{j}$ from [0,1] to (−∞,∞). Other transformation functions such as the logit transformation can also be applied. A prior normal distribution with mean θ is assigned to the probit‐transformed response rate at the lowest dose. The prior variance $\sigma_{1}^{2}$ of $\mu_{1}$ should be sufficiently large to ensure noninformativeness. In practice, θ can simply be set to the probit‐transformed value of the historical control rate ($p_{0}$), that is, $\theta =\Phi^{-1}(p_{0})$. Our sensitivity analysis, reported in the Supporting Information, also confirmed that as long as the prior on $\mu_{1}$ is noninformative (e.g., a relatively large variance $\sigma_{1}^{2}$, such as $\sigma_{1}\geq 2$), the proposed design is robust to various values of θ.

To borrow information across dose levels, a dynamic conditional normal prior is assumed for $\mu_{j}\;|\;\mu_{j-1}$, reflecting our prior belief that the efficacy of adjacent doses is similar and encouraging information sharing across doses. The current model (1) only assumes that the distribution of $\mu_{j}$ is centered around $\mu_{j-1}$ without assuming a monotonic dose–efficacy relationship. However, this model can be adapted to accommodate monotonic dose–efficacy curves by using a truncated normal distribution for $\mu_{j}\;|\;\mu_{j-1}$ with support $\left(\mu_{j-1},\infty \right)$. The variance of this conditional distribution, denoted as $\sigma_{j}^{2}$, quantifies the similarity between adjacent doses, thus controlling the degree of shrinkage or information borrowing. Following Spiegelhalter et al. [29], Gelman [30], and Polson and Scott [31], we assign $\sigma_{j}$ a half‐Cauchy (HC) distribution for stable performance. The location parameter ξ is commonly set as 0. The choice of the scale parameter τ should balance adequate information sharing between doses and the reliability of the dose–response curve estimates. Based on the sensitivity analysis of different τ values presented in the Supporting Information, when J is 2 and the sample size per dose is small (e.g., not exceeding 30), setting $\tau^{2}\in [0.5,5]$ achieves reasonably good performance across a wide range of scenarios. We recommend τ=1 for general use, but users can also perform additional simulations to identify an appropriate value for τ.

Furthermore, once all hyperparameters are specified, we also recommend using the method of moments approach to approximate prior samples from (1) with Beta distributions [32]. Based on the concept of prior effective sample size (PESS) by Morita et al. [33], our design generally performs well when the PESS, using the approximated Beta distribution, falls within (0.01,4), indicating the prior's noninformativeness.

## 3 Trial Design

For a better presentation of the proposed method, we focus on trials with J=2 doses in the following sections and direct readers to the Supporting Information for design details regarding trials with J≥3. Before delving into the design details, we highlight key design differences between trials with J=2 and J≥3 doses. Specifically, we do not impose any nondecreasing dose–efficacy monotonicity for two‐dose trials, allowing for maximum flexibility. However, decision‐making becomes multiplicatively complex when more than two doses are compared. To address this, we impose the nondecreasing dose–efficacy monotonicity for trials involving more than two doses. Additional details can be found in the Supporting Information.

### 3.1 Optimal Dose Definition

Typically, the dose–toxicity relationship in oncology follows a monotonic pattern, where higher doses are associated with an increased likelihood of toxicity. However, this monotonically increasing pattern does not always apply to the dose–efficacy relationship. In some cases, a lower dose may demonstrate similar or even higher efficacy compared to higher doses. Consequently, for many novel cancer treatments, the “less‐is‐more” scenario arises, where a lower dose is as effective or even more effective than a higher dose while causing less toxicity or fewer side effects, thereby offering a more favorable risk‐benefit balance [2]. In this section, we provide a formal definition of dose optimality that accommodates both “more‐is‐better” and “less‐is‐more” scenarios.

Let $\delta_{1}>0$ denote the noninferiority margin and $\delta_{2}>0$ denote the inferiority margin, with the values of $\delta_{1}$ and $\delta_{2}$ being specified from the investigators. In a two‐dose trial, one of the main objectives is to examine whether the lower dose $d_{1}$ maintains efficacy noninferior to the higher dose $d_{2}$ ($p_{2}-p_{j}\leq \delta_{1}$). For example, if $\left(p_{1},p_{2})=(0.4,0.42\right)$, then the difference in efficacy rates between $d_{1}$ and $d_{2}$ is less than $\delta_{1}=0.05$, indicating $d_{1}$ is deemed more desirable in practice than $d_{2}$. As a second example, if $\left(p_{1},p_{2})=(0.1,0.42\right)$, then the improvement in efficacy from $d_{2}$ to $d_{1}$ is greater than $\delta_{2}$, signifying a substantial increase and therefore $d_{2}$ should be preferred than $d_{1}$. It is also possible that $p_{2}-p_{1}$ lies in $\left(\delta_{1},\delta_{2}\right)$; then, we consider this as an inconclusive scenario and suggest seeking factors other than efficacy in the dose selection.

In addition to determining the noninferiority of a lower dose, our design retains the primary objective of conventional phase IIA trials, which is to screen treatments that show promising treatment effects compared to historical controls. Let $p_{0}$ represent the efficacy rate for historical controls, as specified in a conventional phase IIA trial. A dose is deemed futile if its efficacy rate is less than $p_{0}$.

**FIGURE 1:**
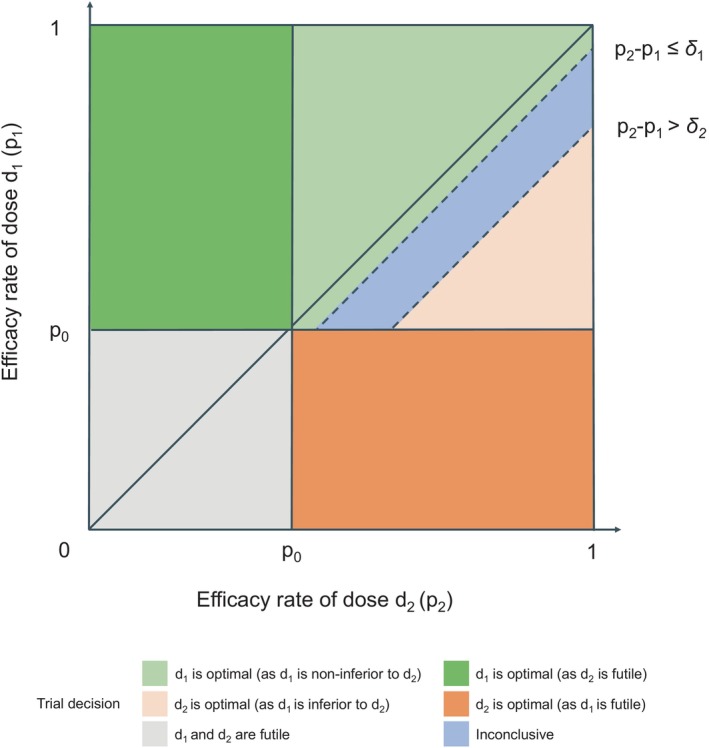
Different types of decisions made by the proposed design, corresponding to the categorization based on the true values.

As detailed in Figure 1, we define the optimality of the lower dose $d_{1}$ as being achieved when both of the following conditions are met: (a) the nonfutility condition, $p_{1}>p_{0}$, meaning the efficacy rate of $d_{1}$ is greater than the historical control rate; and (b) the noninferiority condition, $p_{2}-p_{1}\leq \delta_{1}$ or $p_{2}\leq p_{0}$, meaning the efficacy rate of $d_{1}$ is close to that of $d_{2}$ or $d_{2}$ is futile.

Similarly, the higher dose $d_{2}$ is deemed optimal if the following two conditions are met: (a) the nonfutility condition, $p_{2}>p_{0}$; and (b) the superiority condition, $p_{2}-p_{1}\geq \delta_{2}$ or $p_{1}\leq p_{0}$.

Lastly, if neither dose is futile (i.e., $p_{1}>p_{0}$ and $p_{2}>p_{0}$), the efficacy comparison between $d_{1}$ and $d_{2}$ is considered inconclusive if $p_{2}-p_{1}\in (\delta_{1},\delta_{2})$.

### 3.2 Decision Rules

To determine the optimal dose, we propose a two‐stage design that randomizes patients to the admissible doses at each stage. The randomization can take various approaches, such as simple randomization and outcome‐adaptive randomization. We implement an equal randomization scheme in our paper, allocating $m_{k}$ patients to each active arm in stage k. As a result, in stage 1, a total of $2m_{1}$ patients will be randomized to $d_{1}$ and $d_{2}$. In stage 2, depending on the number of doses passing the per‐dose monitoring described below, the total number of patients enrolled in stage 2 can be 0, $m_{2}$, or $2m_{2}$.

Let ${𝒟}_{k}$ denote the observed data accumulated from the start of the trial to stage k, k=1,2. Based on the data ${𝒟}_{k}$ and the Bayesian dynamic linear model (2), we can obtain the posterior distribution $p_{j}\;|\;{𝒟}_{k}$, j=1,2. The proposed design consists of two major components: per‐dose monitoring in stages 1 and 2, and between‐dose comparison in stage 2.

**(1) Per‐dose monitoring:** At each analysis k, k=1,2, we perform per‐dose futility monitoring to drop the futile dose $d_{j}$ (j=1,2) if the posterior probability of its efficacy rate $p_{j}$ exceeding the historical control rate $p_{0}$ is significantly small, that is,

(2) $$\mathrm{Pr}(p_{j}>p_{0}\;|\;{𝒟}_{k})<a_{k},\;k=1,2$$

where $a_{k}$ is the probability cutoff for the kth analysis. A lower value of $a_{k}$ indicates a more lenient monitoring. For a two‐stage design, we can generally set $a_{1}<a_{2}$ to reflect a more stringent stopping rule as the trial progresses and data accumulates. The values of $a_{1}$ and $a_{2}$ will be optimized according to the calibration procedure outlined in Section 4.

If a dose is deemed futile (i.e., does not pass the per‐dose monitoring) during the interim analysis, no additional patients will be randomized to that dose. If both doses are found to be futile, the trial will stop enrollment, and no dose will be selected. If only one dose passes the per‐dose monitoring at the final analysis, it will automatically be selected as the optimal dose. However, if both doses pass the per‐dose monitoring at the end of stage 2, a subsequent between‐dose noninferiority comparison will be conducted to determine the optimal dose.

**(2) Between‐dose comparison:** We consider a triple‐outcome decision rule for the between‐dose comparison. Compared to the commonly used dichotomous‐outcome decision, which only concludes whether $d_{1}$ is inferior or noninferior to $d_{2}$, the triple‐outcome decision rule allows for a third trial outcome of neither hypothesis being accepted when data suggests that there is a large probability that $d_{2}-d_{1}\in (\delta_{1},\delta_{2})$ or the data is insufficient to make a conclusive decision for $H_{02}$ versus $H_{A2}$ from a statistical standpoint. In such inconclusive cases, the decision is left to clinical judgment based on a comprehensive evaluation of available data, which includes factors such as pharmacokinetics, pharmacodynamics, and other relevant considerations. By allowing a reasonable range of inconclusiveness, the triple‐outcome decision framework facilitates statistical comparison while providing a more realistic basis for decision‐making.

By obtaining the posterior probability of ${\text{PP}}_{\text{NI}}=\mathrm{Pr}(p_{2}-p_{1}<\delta_{1}\;|\;{𝒟}_{2})$, we make three possible trial decisions:

- Select the higher dose $d_{2}$ if $d_{1}$ is inferior to $d_{2}$ as given by $PP_{\mathrm{NI}}\leq c_{1}$;
- Select the lower dose $d_{1}$ if $d_{1}$ is noninferior to $d_{2}$ as given by $PP_{\mathrm{NI}}>c_{2}$;
- Efficacy comparison between $d_{1}$ and $d_{2}$ is inconclusive as given by $c_{1}<PP_{\mathrm{NI}}\leq c_{2}$.

Here, $c_{1}$ and $c_{2}$ ($c_{1}\leq c_{2}$) are probability cutoffs to be calibrated. The difference between $c_{1}$ and $c_{2}$ determines the size of the inconclusive zone, where a larger difference results in a larger zone. Setting $c_{1}=c_{2}$ implies that the triple‐outcome decision‐making reduces to the conventional double‐outcome decision‐making without an inconclusive region. A larger $c_{1}$ indicates that we are more likely to declare that $d_{1}$ is inferior to $d_{2}$. Similarly, a larger $c_{2}$ implies that we are less likely to assert that $d_{1}$ is noninferior to $d_{2}$. In other words, design configurations with larger values of $c_{1}$ and $c_{2}$ are more conservative in accepting a reduction in efficacy, being less likely to select the lower dose.

Incorporating an inconclusive zone provides greater flexibility in determining the optimal dose from multiple perspectives, especially when efficacy data alone are insufficient for a definitive conclusion. By combining the per‐dose monitoring and between‐dose noninferiority comparison criteria, there are four possible trial decisions: (1) both $d_{1}$ and $d_{2}$ are deemed futile and should not be selected, (2) select $d_{1}$, (3) select $d_{2}$, or (4) an inconclusive comparison. In Figure 1, we illustrate these four possible trial decisions based on the dual‐criterion approach.

## 4 Parameter Optimization

We carry out a two‐step calibration procedure to optimize the parameters of the proposed triple‐outcome double‐criterion optimal (TODO) design, including the maximum per‐dose sample size n ($n=m_{1}+m_{2}$), the stage 1 per‐dose sample size $m_{1}$, and the decision cutoffs $a_{1}$, $a_{2}$, $c_{1}$, and $c_{2}$. In Step 1, we determine $\left(n,m_{1},a_{1},a_{2}\right)$ to achieve the desired control of incorrect decision rates for per‐dose monitoring. In Step 2, given the values of $\left(n,m_{1},a_{1},a_{2}\right)$ chosen in Step 1, we identify appropriate values for $c_{1}$ and $c_{2}$ to ensure desirable control of operating characteristics in terms of dose comparison.

Let E represent the event of identifying at least one dose as effective among $d_{1}$ and $d_{2}$. Define the null and alternative hypotheses for monitoring dose $d_{j}$ as $H_{01}(j):p_{j}\leq p_{0}$ and $H_{A1}(j):p_{j}\geq p_{A}$, respectively, where $p_{A}$ is a prespecified promising efficacy rate. The numerical search in Step 1 aims to control $\mathrm{Pr}(E|\cap_{j=1}^{2}H_{01}(j))$ while minimizing the average sample size (ASS) under $\cap_{j=1}^{2}H_{01}(j)$ and maintaining a large $\mathrm{Pr}(E|\cap_{j=1}^{2}H_{A1}(j))$.

The probability $\mathrm{Pr}(E|\cap_{j=1}^{2}H_{01}(j))$ is referred to as the family‐wise type I error rate (FWER) under the global null hypothesis, which posits that all candidate doses are futile. Similarly, $\mathrm{Pr}(E|\cap_{j=1}^{2}H_{A1}(j))$ is referred to as the overall monitoring power (OMP) under the global alternative, representing the probability of identifying at least one promising dose when all doses are effective. When modeling $d_{1}$ and $d_{2}$ independently, the per‐dose monitoring power, Pr(Declare $d_{j}$ effective $\left|H_{A1}(j))(j=1,2\right)$, is calculated as $1-\sqrt{1-\text{OMP}}$. Due to information sharing across doses with the dynamic linear model, the per‐dose monitoring power of the proposed TODO design is expected to exceed $1-\sqrt{1-\text{OMP}}$. This relationship can guide the specification of the target OMP value. For instance, if we target at least 95% OMP with two doses, we can generally expect the per‐dose monitoring power of TODO to be greater than 77.6% (e.g., around 87% as shown in scenario 1.2 of Table 1).

**TABLE 1:** Simulation results for the two‐dose trials, with optimal doses and correct selection decisions highlighted in boldface. Selection %: Percentage of selecting the dose in the final analysis. SIR: Percentage of having an inconclusive result. IDR: Rate of incorrect decisions. WL: Weighted loss (with $w_{l}=0.40$). ASS: Average sample size. Go %: Percentage of making a “go” decision in the per‐dose monitoring, that is, the per‐dose monitoring power. FWER/OMP %: Percentage of trials identifying at least one dose as admissible in the per‐dose futility monitoring, which represents the family‐wise type I error rate (FWER) in Scenario 1.1 and the overall monitoring power (OMP) in the other scenarios.

	Selection %					ASS		Go %		
Method	$d_{1}$	$d_{2}$	SIR	IDR	WL	$d_{1}$	$d_{2}$	$d_{1}$	$d_{2}$	FWER/OMP %
Scenario 1.1: $\left(p_{1},p_{2})=(0.20,0.20\right)$										
TODO	4.2	4.7	0.0	8.9	8.9	18.5	21.8	4.2	5.6	8.9
TODO‐BB	4.9	4.8	0.0	9.7	9.7	21.7	21.8	4.9	5.0	9.7
DREAMM‐2	4.4	4.7	0.0	9.1	9.1	21.8	21.9	4.5	4.8	9.1
Scenario 1.2: $\left(p_{1},p_{2})=(\text{0.40},0.40\right)$										
TODO	**67.6**	22.1	5.6	26.8	29.0	28.2	28.5	85.4	87.2	95.3
TODO‐BB	**61.2**	24.4	9.3	29.5	33.2	28.0	28.1	77.1	77.4	94.9
DREAMM‐2	**51.7**	43.4	0.0	48.3	48.3	28.1	28.2	76.4	77.7	95.1
Scenario 1.3: $\left(p_{1},p_{2})=(\text{0.40},0.45\right)$										
TODO	**57.0**	32.4	8.3	34.7	38.0	28.5	28.8	86.9	94.9	97.8
TODO‐BB	**49.5**	34.6	13.5	36.9	42.4	28.0	28.5	77.1	89.7	97.7
DREAMM‐2	**38.6**	59.0	0.0	61.4	61.4	28.1	28.5	77.1	90.1	97.6
Scenario 1.4: $\left(p_{1},p_{2})=(0.40,\text{0.60}\right)$										
TODO	17.0	**75.4**	7.5	17.1	20.1	28.7	29.0	87.4	99.9	99.9
TODO‐BB	14.1	**73.8**	11.9	14.3	19.0	28.0	29.0	77.1	99.6	99.9
DREAMM‐2	8.0	**91.9**	0.0	8.1	8.1	28.1	29.0	77.4	99.7	99.9
Scenario 1.5: $\left(p_{1},p_{2})=(0.20,\text{0.40}\right)$										
TODO	5.3	**72.6**	1.2	26.2	26.7	24.4	28.0	10.4	78.4	79.1
TODO‐BB	2.9	**74.6**	1.0	24.4	24.8	21.7	28.1	4.9	77.4	78.5
DREAMM‐2	2.1	**77.0**	0.0	23.0	23.0	21.9	28.1	5.0	78.0	79.0
Scenario 1.6: $\left(p_{1},p_{2})=(0.40,\text{0.70}\right)$										
TODO	3.6	**93.7**	2.7	3.7	4.7	28.6	29.0	87.0	100.0	100.0
TODO‐BB	2.8	**92.4**	4.8	2.8	4.7	28.0	29.0	77.1	100.0	100.0
DREAMM‐2	1.3	**98.7**	0.0	1.3	1.3	28.1	29.0	77.3	100.0	100.0
Scenario 1.7: $\left(p_{1},p_{2})=(\text{0.40},0.43\right)$										
TODO	**61.6**	28.1	7.3	31.1	34.0	28.4	28.7	86.4	92.4	97.0
TODO‐BB	**54.7**	30.6	11.7	33.7	38.3	28.0	28.4	77.1	86.0	96.9
DREAMM‐2	**44.4**	52.4	0.0	55.6	55.6	28.1	28.4	76.5	86.0	96.8
Scenario 1.8: $\left(p_{1},p_{2})=(\text{0.45},0.40\right)$										
TODO	**80.3**	11.7	5.2	14.4	16.5	28.5	28.6	93.3	88.2	97.3
TODO‐BB	**76.2**	13.3	8.2	15.7	18.9	28.5	28.1	89.6	77.4	97.7
DREAMM‐2	**68.1**	29.4	0.0	31.9	31.9	28.6	28.1	89.8	76.8	97.5
Scenario 1.9: $\left(p_{1},p_{2})=(\text{0.60},0.40\right)$										
TODO	**97.0**	0.8	0.7	2.3	2.6	28.7	28.7	98.4	87.8	98.5
TODO‐BB	**97.3**	0.8	1.8	0.9	1.6	29.0	28.1	99.7	77.4	99.9
DREAMM‐2	**95.4**	4.6	0.0	4.6	4.6	29.0	28.1	99.8	76.9	99.9

The numerical search in Step 1 involves conducting simulation studies to compute $\mathrm{Pr}(E|\cap_{j=1}^{2}H_{01}(j))$ and $\mathrm{Pr}(E|\cap_{j=1}^{2}H_{A1}(j))$ for each possible combination of $\left(m_{1},a_{1},a_{2}\right)$ across a prespecified grid ($m_{1}<n$, $a_{1}<a_{2}$) for a given value of n. Among the admissible values of $\left(m_{1},a_{1},a_{2}\right)$ that control the FWER at a nominal level $\alpha_{1}$ (e.g., $\alpha_{1}=10\%$), the optimal combination is identified as the one that minimizes the ASS under the global null while ensuring the OMP is within 1% of the maximum OMP achieved. For example, given a maximum OMP of 90%, any OMP of 89% or higher is acceptable. The motivation is to select the combination of $\left(m_{1},a_{1},a_{2}\right)$ that achieves a higher early stopping rate under the global null while keeping the OMP as close as possible to the maximum OMP under the global alternative. For each possible value of n, we identify the optimal combination of $\left(m_{1},a_{1},a_{2}\right)$. Subsequently, we determine the smallest n that meets the target OMP level $\beta_{1}$ (e.g., $\beta_{1}=90\%$).

Step 1 can be considered a generalization of the calibration process used in the Bayesian optimal phase 2 (BOP2) design [34], which aims to maximize power while controlling the type I error rate for phase II single‐arm futility or toxicity monitoring. Following the BOP2 design, we impose a regularization such that $a_{1}=a_{2}{\left(m_{1}/n\right)}^{\lambda }$ (0<λ≤1). Consequently, searching for $\left(a_{1},a_{2}\right)$ becomes equivalent to searching for $\left(\lambda ,a_{2}\right)$. This ensures that $a_{1}$ depends on the stage 1 sample size $m_{1}$: the smaller the stage 1 sample size, the less likely it is to prematurely stop an arm, thereby reducing the risk of erroneous stopping due to sparse data.

After an optimal combination of $\left(n,m_{1},a_{1},a_{2}\right)$ is identified, we perform Step 2 of the numerical search to identify the optimal values of $c_{1}$ and $c_{2}$. We are particularly interested in the two special sets of alternative hypotheses, where both candidate doses are more effective than the historical control. In the first alternative $H_{A}^{*}:(p_{1},p_{2})=(p_{A},p_{A}+\delta_{1})$, the low dose $d_{1}$ is noninferior to the high dose $d_{2}$; in the second alternative $H_{A}^{+}:(p_{1},p_{2})=(p_{A},p_{A}+\delta_{2})$, the low dose is inferior to the high dose. As $c_{1}$ and $c_{2}$ are probability cutoffs for the between‐dose comparison, choosing alternative scenarios in which all candidate doses show promise can minimize the potential masking effects of per‐dose monitoring. In Step 2, we consider the following three key performance metrics:

- The size of the inconclusive region (SIR), defined as the probability of having an inconclusive trial outcome averaged across $H_{A}^{*}$ and $H_{A}^{+}$.
- The maximum rate of selecting an inadequate dose (MRID), defined as the maximum probability of incorrectly selecting a lower inferior dose across $H_{A}^{+}$, that is, $\mathrm{Pr}(\text{Select}\;d_{1}\;|\;H_{A}^{+})$.
- The weighted loss (WL), defined as

$$\text{WL}=\text{IDR}+w_{l}\text{SIR}$$

where IDR denotes the average incorrect decision rate under $H_{A}^{*}$ and $H_{A}^{+}$, and $0\leq w_{l}\leq 1$ is the discount factor of an inconclusive decision toward the incorrect decision. The incorrect decisions include selecting a higher dose (i.e., an overdose) under $H_{A}^{*}$, selecting a lower inferior dose (i.e., an inadequate dose) under $H_{A}^{+}$, or dropping all candidate doses when there exists an optimal dose, When two doses are compared, then the IDR $=0.5\mathrm{Pr}(\text{Select}\;d_{2}\;\text{or none}\;|\;H_{A}^{*})+0.5\mathrm{Pr}(\text{Select}\;d_{1}\;\text{or none}\;|\;H_{A}^{+})$. When certain types of incorrect dose–selection decisions (e.g., selecting a lower dose under $H_{A}^{+}$) are more critical than others, this definition of the IDR can be generalized using a weighted average approach, assigning greater penalties to more serious incorrect decisions (e.g., assigning more weight to $\mathrm{Pr}(\text{Select}\;d_{1}\;\text{or none}\;|\;H_{A}^{+})$).

The definition of IDR does not account for inconclusive results, as such outcomes provide an opportunity to evaluate the study doses using additional data and do not eliminate the possibility of selecting the true optimal dose. Consequently, an inconclusive result may be more acceptable than selecting a suboptimal dose. However, it is not as desirable as making a correct dose selection. For this reason, we discount the impact of the SIR in the WL function. The magnitude of the discount is quantified by the weighted value, $w_{l}$, which needs to be specified prior to starting a trial. A larger value of $w_{l}$ indicates a greater penalty for making an inconclusive decision, suggesting that a definitive outcome based on efficacy data is more expected, rather than an inconclusive one. Otherwise, a lower value of $w_{l}$ is more appropriate. The specification of $w_{l}$ depends on multiple aspects, including the investigator's preference and the sample size. For example, setting $w_{l}=1$ implies that making an inconclusive decision is the same as making a definitive incorrect dose–selection decision. Conversely, setting $w_{l}=0$ indicates that an inconclusive result is fully acceptable. The impact of the $w_{l}$ will be discussed in detail in Section 5.3 through simulation studies.

Given a grid of possible values of $c_{1}$ and $c_{2}$ ($c_{1}\leq c_{2}$), the optimal $c_{1}$ and $c_{2}$ are identified by minimizing the WL under the following two hard constraints:

(a) The MRID should be controlled at a given value $\alpha_{2}$, say $\alpha_{2}\in [10\%,25\%]$. In practice, the study doses are selected based on prior phase I trials, suggesting that their toxicity rates are expected to be acceptable, with treatment efficacy carrying more weight in decision‐making. Setting a hard constraint for MRID indicates that choosing an inadequate and inferior dose under $H_{A}^{+}$ is typically considered more unacceptable than selecting a higher dose under $H_{A}^{*}$, provided that both doses are safe and tolerable. Therefore, this constraint serves as an additional safeguard to control the risk of choosing an inadequate dose.

(b) The SIR should be controlled at a given value $\alpha_{3}$, say $\alpha_{3}\in [0\%,20\%]$. As the inconclusive region allows for the incorporation of various data types in selecting the optimal dose, setting an upper bound for the SIR ensures that efficacy outcomes remain the dominant factor in dose optimization. Moreover, without any constraint on the SIR, the design may consistently result in inconclusive outcomes, which would undermine the decision‐making process.

Algorithm 1 details the two‐step parameter optimization process for two‐dose trials. The generalized version of this algorithm for multidose trials is provided in the Supporting Information. Employing such a two‐step calibration approach, the TODO design can effectively balance the identification of optimal doses across various scenarios, thereby offering more effective and meaningful operating characteristics for dose optimization.

It is important to note that the sample size required for the TODO design is generally much smaller than what would be needed for a formal noninferiority trial [35]. This is because our sample size determination is based on per‐dose monitoring criteria (i.e., in Step 1 of Algorithm 1) rather than on powering a formal noninferiority between‐dose comparison. Even for dose comparison in the final analysis, the TODO design focuses on minimizing weighted loss and controlling metrics such as MRID and SIR rather than strictly adhering to the traditional type I and type II error controls. Furthermore, in conventional noninferiority testing, the noninferiority and inferiority margins $\left(\delta_{1},\delta_{2}\right)$ are typically set with $\delta_{1}=0$ and $\delta_{2}$ as a small value (e.g., $\delta_{2}=0.05$). While this approach is more rigorous, it is not recommended for early‐phase, multidose randomized trials due to the larger sample size required. In the TODO design, we assume relatively larger values for $\delta_{1}$ and $\delta_{2}$ (e.g., $\delta_{1}=0.05$ and $\delta_{2}=0.2$) to provide a more preliminary assessment of dose noninferiority. As a result, the TODO design is specifically tailored for small‐to‐moderate sample sizes, making it significantly more feasible for early‐phase dose optimization trials.

ALGORITHM 1 **Parameter optimization algorithm for $\left(n,m_{1},a_{1},a_{2},c_{1},c_{2}\right)$.**

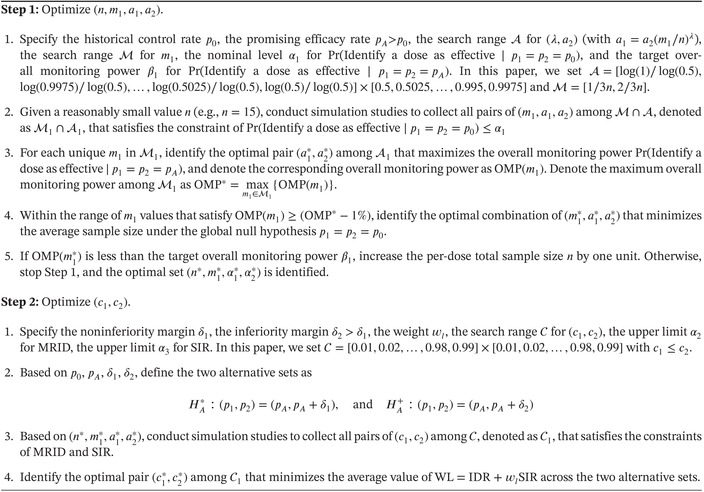

## 5 Numerical Studies

### 5.1 Numerical Illustration

Consider a two‐dose trial where the efficacy rates under the null and alternative hypotheses are $p_{0}=0.2$ and $p_{A}=0.4$, respectively, and the noninferiority and inferiority margins for the between‐dose comparison are $\delta_{1}=0.05$ and $\delta_{2}=0.2$. We set the hyperparameters in the Bayesian dynamic linear model (1) as $\theta =\Phi^{-1}(p_{0})$, $\sigma_{1}=3$, ξ=0, and τ=1. This specification results in a prior effective sample size (PESS) [33] of 2 for $d_{1}$ and less than 1 for $d_{2}$, indicating prior noninformativeness. To optimize the design parameters, we specify the following reasonable values in Algorithm 1: $\alpha_{1}=10\%$, $\beta_{1}=95\%$, $\alpha_{2}=20\%$, $\alpha_{3}=15\%$, and $w_{l}=0.40$. As discussed in Section 4, with a target OMP of $\beta_{1}=95\%$, the per‐dose monitoring power of the TODO design is expected to exceed 77.6% (e.g., around 87% as shown in scenario 1.2 of Table 1), which is confirmed in the following simulation studies. In contrast, using Simon's optimal two‐stage design [36] with a per‐dose type I error rate of 5.19% (e.g., FWER ≈10%), a maximum sample size of n=46 per dose is required to achieve the same per‐dose monitoring power (i.e., 87%) as the TODO design.

**FIGURE 2:**
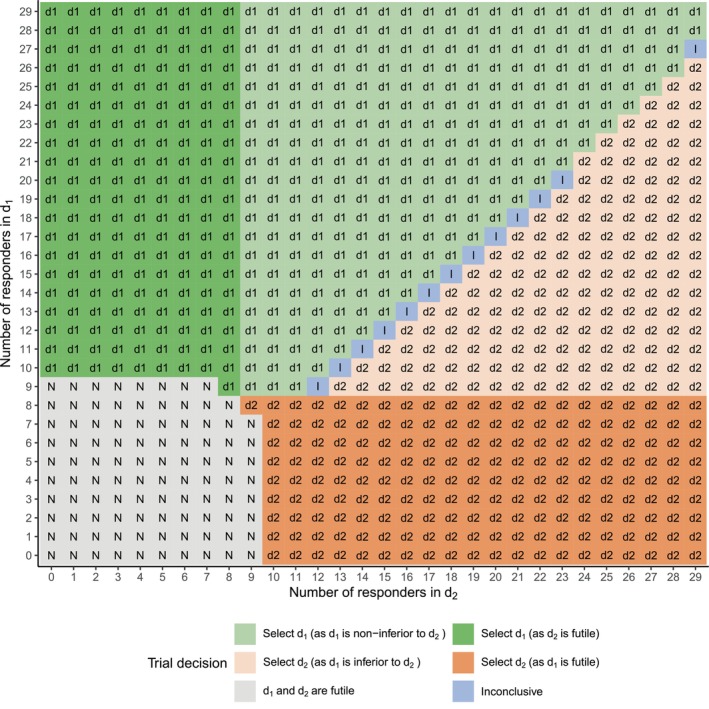
Final analysis decision plot based on the number of efficacy responders in the proposed design, assuming both doses pass the per‐dose monitoring during the interim analysis, for the two‐dose trial described in Section 5.1.

Based on Algorithm 1, the optimal maximum sample size is determined to be n=29, with a stage 1 sample size of $m_{1}=10$. The optimized decision cutoffs $a_{1}$, $a_{2}$, $c_{1}$, and $c_{2}$ are summarized in Table S1 of the Supporting Information. For a two‐dose trial, it is feasible to enumerate all possible efficacy outcomes for $d_{1}$ and $d_{2}$ given fixed n and $m_{1}$ and then apply the proposed model and decision rules to each outcome combination. In Figure 2, we present the final analysis decision plot for the TODO design, assuming both doses pass the per‐dose monitoring during the interim analysis. The interim analysis decision plot and the final analysis decision plots for scenarios where only one dose passes the per‐dose monitoring during the interim analysis, are provided in Figures S1–S3 in the Supporting Information. In practice, these decision plots can be pre‐generated before trial enrollment begins and used explicitly during the conduct of the trial.

### 5.2 Main Simulation Results

In the main paper, we report simulation results of the TODO design under nine scenarios involving two doses. As shown in Table 1, these nine scenarios cover a wide range of potential scenarios that are relevant to the dose monitoring and optimization trials. In particular, scenarios 1.1 (the global null) and 1.2 are used to optimize the sample sizes and cutoffs $a_{1}$ and $a_{2}$ for per‐dose monitoring, while scenarios 1.3 ($H_{A}^{*}$) and 1.4 ($H_{A}^{+}$) are employed to optimize $c_{1}$ and $c_{2}$ for between‐dose comparisons. In scenarios 1.2, 1.3, and 1.7, $d_{1}$ is noninferior to $d_{2}$, thereby making $d_{1}$ the optimal dose. Conversely, $d_{2}$ is optimal in scenarios 1.4–1.6 due to its superior efficacy. Scenarios 1.8 and 1.9 exhibit a decreasing trend in efficacy with the increasing dose, thereby favoring the lower dose $d_{1}$. Through simulation studies, we compare the TODO design with two alternative approaches, both based on independent Beta‐Binomial modeling with a $\text{Beta}(p_{0},1-p_{0})$ prior for the two doses but differing in their design details. For a fair comparison, both alternative approaches use the same maximum sample size n=29 and stage 1 sample size $m_{1}=10$ as the TODO design.

The first approach is an unoptimized design inspired by the DREAMM‐2 trial (referred to as DREAMM‐2). This design also employs a combined per‐dose monitoring and between‐dose comparison approach but lacks systematic optimization and does not include an inconclusive trial decision option. Specifically, according to the statistical analysis plan available at https://cdn.clinicaltrials.gov/large‐docs/78/NCT03525678/Prot_002.pdf, the interim futility cutoff is determined by controlling the early stopping rate under $H_{A1}(j)$ at approximately 5%, while the final futility cutoff is determined by controlling the FWER at 10% under $\cap_{j=1}^{2}H_{01}(j)$. For dose comparison, the DREAMM‐2 design selects the higher dose if $\mathrm{Pr}(p_{2}>p_{1}|{𝒟}_{2})\geq c^{\prime }$; otherwise, it selects the lower dose. In this paper, $c^{\prime }=0.6$ is used to ensure comparability with other methods. It is worth noting that the target early stopping rate of 5% and the design cutoff $c^{\prime }=0.6$ for the DREAMM‐2 design are heuristically selected, whereas the TODO design employs a systematic rule to optimize these parameters.

The second design (referred to as TODO‐BB) is a simplified version of the proposed TODO design, differing only in its use of independent dose modeling instead of dynamic linear modeling. The decision cutoffs of TODO‐BB are also optimized using Algorithm 1. The parameters for the DREAMM‐2 and TODO‐BB designs are summarized in Tables S2 and S3 of the Supporting Information, respectively.

The operating characteristics of the TODO, TODO‐BB, and DREAMM‐2 designs are computed based on 10,000 trial replications and reported in Table 1. The proposed TODO design shows the most promising overall performance, particularly in scenarios where the lower dose is optimal. Due to the calibrated cutoffs, all three designs maintain the FWER below $\alpha_{1}=10\%$ in scenario 1.1. The utilization of the more efficient dynamic linear model (DLM) in the TODO design demonstrates its advantage in enhancing per‐dose monitoring power. In scenarios 1.2–1.9, the DLM‐based TODO design also achieves a higher percentage of “go” decisions for promising doses ($p_{j}>p_{0}$) compared to the other two approaches.

In scenarios 1.2, 1.3, and 1.7–1.9, where $d_{1}$ is optimal, the TODO design is more effective at detecting promising doses and correctly selecting the optimal one compared to the other two designs, resulting in the lowest weighted loss. In scenarios where the higher dose is better (scenarios 1.4–1.6), the correct selection percentages (i.e., the frequency of selecting the optimal dose) of the TODO and TODO‐BB designs are comparable. The DREAMM‐2 method excels in identifying the optimal dose in scenarios 1.4 and 1.6. It could be argued that the TODO design can be calibrated to be more aggressive by choosing a larger $w_{l}$, as shown in the next section. However, this comes at the expense of reduced flexibility in leveraging other data types for dose optimization. In scenario 1.1, the TODO design achieves the smallest ASS, indicating their higher efficiency in identifying futile doses. Across the remaining scenarios, the three methods show comparable ASS.

Furthermore, we report the average bias and mean squared error (MSE) across all doses to evaluate the efficiency and robustness of the TODO and TODO‐BB designs in estimating efficacy rates for $d_{1}$ and $d_{2}$. Let ${\hat{p}}_{j}$ denote the posterior mean estimate of the efficacy rate $p_{j}$ at dose $d_{j}$. The average bias and MSE are calculated by averaging the bias and MSE values across all doses as follows:

$$\begin{array}{ll} \text{Average Bias} & =\frac{\sum_{j=1}^{2}({\hat{p}}_{j}-p_{j})}{2},\\ \text{Average MSE} & =\frac{\sum_{j=1}^{2}{\left({\hat{p}}_{j}-p_{j}\right)}^{2}}{2} \end{array}$$

**FIGURE 3:**
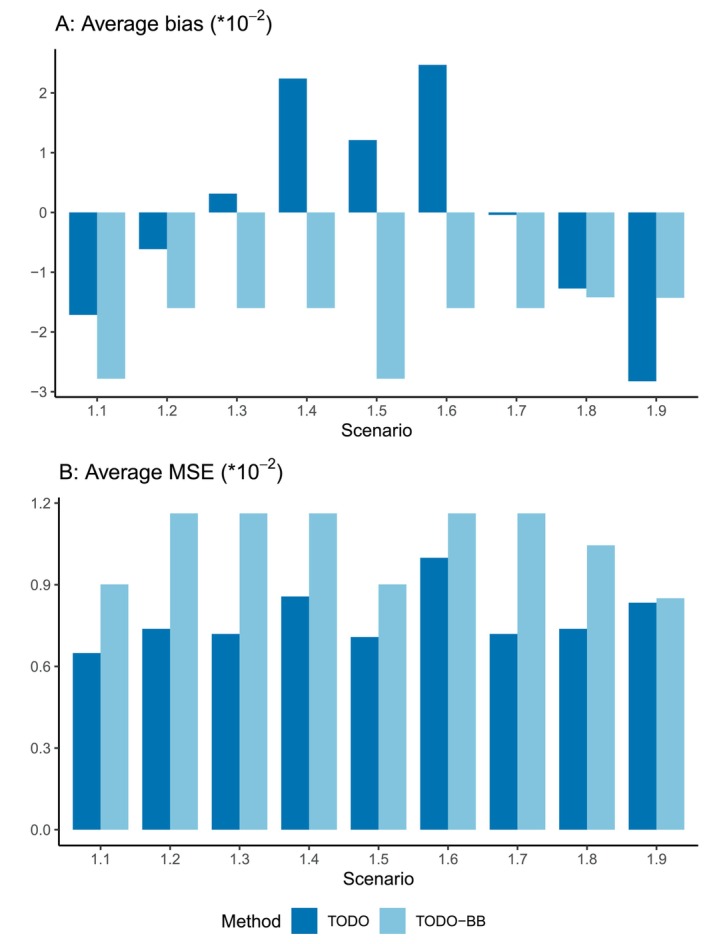
The average bias and mean squared error (MSE) across all doses for the proposed TODO design and the design using a beta‐binomial model (TODO‐BB) with a sample size of 29.

As illustrated in Figure 3, the TODO design consistently demonstrates a comparable average bias to the TODO‐BB design. Specifically, the bias of the TODO design falls within the range of [−0.03, 0.03]. Notably, the TODO design exhibits lower average MSE in most scenarios, indicating its superior efficiency and robustness in estimating dose–efficacy relationships.

### 5.3 Exploring the Impact of $w_{l}$

**FIGURE 4:**
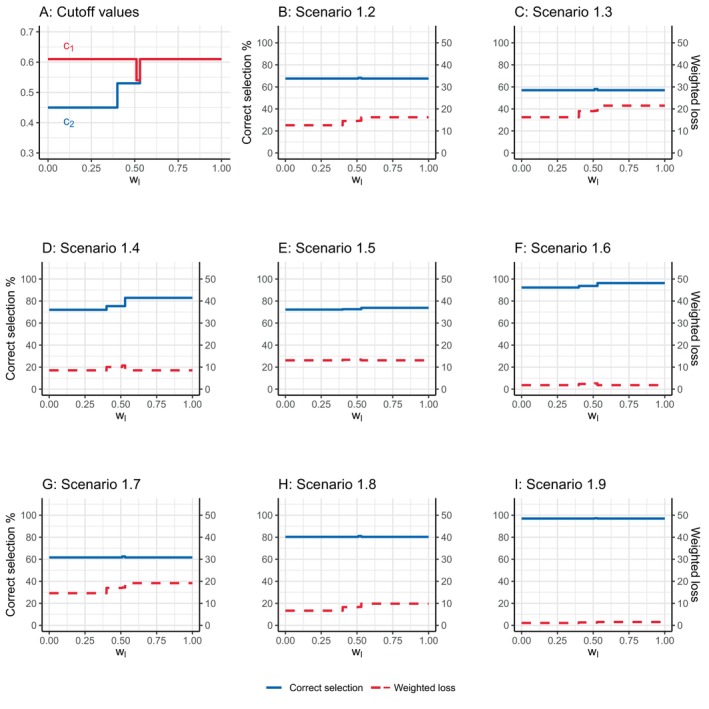
The influence of $w_{l}$ on the calibrated cutoff values of the proposed design (panel A) and the selection percentage of the optimal dose (solid lines in panels B–I) as well as the weighted loss (dashed lines in panels B–I) with a sample size of 29.

As the value of $w_{l}$ plays a critical role in determining the values of $c_{1}$ and $c_{2}$ in between‐dose comparisons, we examine the impact of $w_{l}$ on the design's performance by varying $w_{l}$ from 0 to 1 in scenarios 1.2 to 1.9. As illustrated in Figure 4 panel (A), the gap between $c_{1}$ and $c_{2}$ decreases with a higher $w_{l}$. When $w_{l}=1$, $c_{1}$ and $c_{2}$ converge to an identical value. Since the gap between $c_{1}$ and $c_{2}$ determines the SIR, an increased value of $w_{l}$, in turn, decreases the SIR. Furthermore, it is observed that the value of $c_{1}$ exhibits more pronounced changes with respect to the changes in $w_{l}$. This indicates that with a larger $w_{l}$, the TODO design is more likely to declare the inferiority of the lower dose, leading to a greater selection of the higher dose.

The impact of $w_{l}$ on the design operating characteristics through its direct effect on $c_{1}$ and $c_{2}$ is reflected in Figure 4 panels B to I. Particularly, in scenarios 1.4 to 1.6, where the high dose is the optimal one, the increasing trend of correct selection percentage aligns with the changes in $c_{1}$. Figures S4 and S5 in the Supporting Information present a similar pattern for $w_{l}$ with sample sizes of 36 and 91, respectively. These simulation results elucidate how $w_{l}$ influences the operating characteristics of the proposed design, which is crucial for effectively designing and conducting the trial to ensure its successful execution.

### 5.4 Additional Simulation Studies

Under scenarios 1.1–1.9, we investigate the impact of the prior mean specification for the lowest dose θ and the scale parameter $\tau^{2}$ to assess the robustness of the proposed design in the Supporting Information. Furthermore, we report additional simulation studies under different trial configurations. These include studies with varying FWER and OMP requirements, such as $\left(\alpha_{1},\beta_{1})=(5\%,95.0\%\right)$ and $\left(\alpha_{1},\beta_{1})=(1\%,99.8\%\right)$, which result in larger maximum sample sizes of 36 and 91 per dose arm, respectively. We also consider studies with different hypothesis specifications, such as $\left(p_{0},p_{A})=(0.1,0.3\right)$ or $\left(p_{0},p_{A})=(0.3,0.5\right)$, as well as studies for trials involving three doses. Overall, the proposed TODO design consistently demonstrates superior performance in these additional simulation studies.

## 6 Simultaneous Evaluation of Toxicity and Efficacy

### 6.1 Utility‐Based Risk‐Benefit Tradeoff

The proposed trial design is versatile and can be tailored to meet specific trial needs. We illustrate adapting the TODO design to simultaneously evaluate toxicity and efficacy and determine the optimal dose using a utility function for risk‐benefit trade‐offs. Denote the true toxicity rate (e.g., the probability of having a grade ≥3 treatment‐related adverse event) of dose $d_{j}$ by $q_{j}$, and define the utility function as

$$u_{j}=p_{j}-\rho q_{j}$$

where the weight ρ reflects the clinical preference for balancing efficacy and toxicity. A larger ρ indicates a greater penalty for toxicity. Let $q_{0}$ denote the upper limit of the toxicity rate, $q_{A}$ the lower acceptable toxicity rate, and $\delta_{1}$ (or $\delta_{2}$) the noninferiority (or inferiority) margin in terms of utility.

Similar to Section 3.1, we define the optimality of the lower dose $d_{1}$ as being achieved when both of the following conditions are met: (a) the admissibility condition, $q_{1}<q_{0}$ (safe) and $p_{1}>p_{0}$ (effective); (b) the noninferiority condition, $u_{2}-u_{1}\leq \delta_{1}$ or $d_{2}$ is inadmissible with $q_{2}\geq q_{0}$ or $p_{2}\leq p_{0}$. Similarly, the higher dose $d_{2}$ is deemed optimal if the following two conditions are met: (a) the admissibility condition, $q_{2}<q_{0}$ (safe) and $p_{2}>p_{0}$ (effective); and (b) the superiority condition, $u_{2}-u_{1}\geq \delta_{2}$ or $d_{1}$ is inadmissible. Lastly, if both doses are admissible, the utility comparison between $d_{1}$ and $d_{2}$ is considered inconclusive if $u_{2}-u_{1}\in (\delta_{1},\delta_{2})$.

We utilize the same modeling and decision‐making strategies outlined in Sections 2 and 3 for evaluating both efficacy and toxicity. While the association between toxicity and efficacy can be further incorporated into the Bayesian dynamic linear model, we have not found this to be beneficial to the design's performance. For the per‐dose monitoring, a dose $d_{j}$ should be dropped if it is either overly toxic or futile as given by $\mathrm{Pr}(q_{j}<q_{0}\;|\;{𝒟}_{k})<a_{k},$ or $\mathrm{Pr}(p_{j}>p_{0}\;|\;{𝒟}_{k})<a_{k},$ k=1,2. At the end of the trial when both $d_{1}$ and $d_{2}$ remain admissible, the between‐dose comparisons are made based on the posterior probability of PP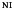 $=\mathrm{Pr}(u_{2}-u_{1}<\delta_{1}\;|\;{𝒟}_{2})$.

To calibrate the design parameters, we follow the same procedure described in Section 4. More specifically, to calculate the FWER and OMP in Step 1, the global null is defined as $\cap_{j=1}^{2}\{H_{01}(j)\cap H_{02}(j)\}$ with $H_{02}(j):q_{j}\geq q_{0}$ and the global alternative is $\cap_{j=1}^{2}\{H_{A1}(j)\cap H_{A2}(j)\}$ with $H_{A2}(j):q_{j}\leq q_{A}$. In Step 2 to optimize $c_{1}$ and $c_{2}$, assuming all candidate doses are safe with $q_{j}=q_{A}$, we construct the two alternatives primarily based on the efficacy rate. In particular, the first alternative is constructed as ${\tilde{H}}_{A}^{*}=H_{A}^{*}\cap \{q_{1}=q_{2}=q_{A}\}$, and the second one is ${\tilde{H}}_{A}^{+}=H_{A}^{+}(j)\cap \{q_{1}=q_{2}=q_{A}\}$, where $H_{A}^{*}$ and $H_{A}^{+}$ are defined in Section 4. Therefore, $d_{1}$ is optimal under ${\tilde{H}}_{A}^{*}$ and $d_{2}$ is optimal under ${\tilde{H}}_{A}^{+}$, respectively.

### 6.2 Further Simulation Studies

We evaluate the operating characteristics of the generalized TODO design for simultaneously assessing toxicity and efficacy under 15 scenarios, as shown in Table 2. The specification of the hyperparameters for the toxicity model follows the same rule used for the efficacy model in Section 5.1. We consider a two‐dose trial by setting $p_{0}=0.25$, $p_{A}=0.4$, $q_{0}=0.4$, $q_{A}=0.2$, $\delta_{1}=0.05$, and $\delta_{2}=0.2$, respectively. For the utility function, we use ρ=0.67 to balance toxicity and efficacy, indicating that each unit increase in the toxicity rate should be offset by a 0.67 unit increase in the efficacy rate. To maintain the same level of $\alpha_{1}=10\%,\alpha_{2}=20\%,\alpha_{3}=15\%$ and $\beta_{1}=95\%$ with $w_{l}=0.40$ as in Section 5.1, the maximum sample size per dose arm is n=26 patients, and an interim analysis for per‐dose monitoring is conducted after enrolling $m_{1}=11$ patients per arm. The other design parameters are provided in Table S1 of the Supporting Information.

**TABLE 2:** Scenarios 2.1–2.15 for simultaneously evaluating toxicity and efficacy. Optimal doses are highlighted in boldface.

	Dose $d_{1}$			Dose $d_{2}$			Utility
Scenario	Toxicity rate	Efficacy rate	Utility	Toxicity rate	Efficacy rate	Utility	difference
2.1	0.40	0.20	−0.07	0.40	0.20	−0.07	0.00
2.2	**0.25**	**0.40**	**0.23**	0.25	0.40	0.23	0.00
2.3	0.25	0.40	0.23	**0.25**	**0.60**	**0.43**	0.20
2.4	**0.25**	**0.40**	**0.23**	0.25	0.45	0.28	0.05
2.5	0.30	0.20	0.00	0.35	0.20	−0.03	−0.03
2.6	0.40	0.25	−0.02	0.50	0.30	−0.04	−0.02
2.7	0.15	0.20	0.10	**0.20**	**0.40**	**0.27**	0.17
2.8	0.15	0.40	0.30	**0.20**	**0.65**	**0.52**	0.22
2.9	0.15	0.50	0.40	**0.20**	**0.75**	**0.62**	0.22
2.10	**0.20**	**0.40**	**0.27**	0.40	0.60	0.33	0.07
2.11	**0.15**	**0.40**	**0.30**	0.20	0.45	0.32	0.02
2.12	**0.05**	**0.40**	**0.37**	0.20	0.50	0.37	0.00
2.13	**0.05**	**0.50**	**0.47**	0.20	0.60	0.47	0.00
2.14	**0.20**	**0.50**	**0.37**	0.45	0.40	0.10	−0.27
2.15	**0.15**	**0.50**	**0.40**	0.20	0.45	0.32	−0.08

Table 3 summarizes the simulation results from 10,000 simulated trials for each scenario. The proposed method effectively eliminates overly toxic or inactive doses in scenarios 2.1, 2.5, and 2.6, where no optimal dose exists. Across all scenarios, it successfully controls both the size of the inconclusive region and the rate of selecting an inadequate dose. The results further demonstrate that the proposed TODO design more frequently selects the correct optimal dose in various scenarios. In Table S4 of the Supporting Information, additional simulation results with a sample size of 33 per dose arm also clearly show that the performance of the generalized TODO design improves with increasing sample size.

**TABLE 3:** Simulation results for simultaneously evaluating toxicity and efficacy, with optimal doses and correct selection decisions highlighted in boldface. Selection %: Percentage of selecting the dose in the final analysis. SIR: Percentage of having an inconclusive result. IDR: Rate of incorrect decisions. WL: Weighted loss (with $w_{l}=0.40$). ASS: Average sample size. Go %: Percentage of making a “go” decision in the futility monitoring, that is, the per‐dose monitoring power. FWER/OMP %: Percentage of trials identifying at least one dose as effective in the futility monitoring, which represents the family‐wise type I error rate (FWER) in Scenario 2.1 and the overall monitoring power (OMP) in the other scenarios.

	Selection %					ASS		Go %		
Scenario	$d_{1}$	$d_{2}$	SIR	IDR	WL	$d_{1}$	$d_{2}$	$d_{1}$	$d_{2}$	FWER/OMP %
2.1	5.2	4.3	0.3	9.8	9.9	17.6	17.5	5.7	5.5	9.8
2.2	**55.2**	26.1	14.1	30.7	36.4	25.4	25.3	86.8	85.6	95.4
2.3	19.5	**66.1**	11.2	22.8	27.2	25.4	25.6	87.1	89.5	96.7
2.4	**45.6**	35.6	15.0	39.5	45.5	25.4	25.4	87.1	87.9	96.1
2.5	12.8	7.1	0.7	20.6	20.9	19.4	18.7	14.1	11.0	20.6
2.6	11.0	2.3	0.2	13.5	13.5	19.1	17.1	11.4	3.1	13.4
2.7	12.5	**68.3**	7.5	24.2	27.2	23.0	25.0	37.5	86.5	88.2
2.8	14.0	**74.8**	11.0	14.3	18.7	25.7	25.9	95.7	97.2	99.7
2.9	14.0	**75.5**	10.3	14.2	18.3	25.9	25.9	98.9	97.3	99.9
2.10	**64.6**	21.7	6.2	29.2	31.6	25.4	23.4	90.6	38.0	92.5
2.11	**56.2**	27.1	16.0	27.8	34.2	25.7	25.7	96.0	95.4	99.3
2.12	**62.5**	24.0	13.3	24.3	29.6	25.8	25.8	96.5	96.2	99.7
2.13	**63.4**	23.3	13.3	23.3	28.6	26.0	25.9	99.7	96.9	100.0
2.14	**91.5**	1.0	0.8	7.7	8.0	25.6	21.7	92.8	19.8	93.2
2.15	**78.8**	10.6	10.4	10.8	15.0	25.9	25.7	99.2	95.8	99.8

## 7 Concluding Remarks

The proposed TODO design offers an efficient tool for identifying the optimal dose in randomized multidose trials. We employ a double‐criterion approach to evaluate the efficacy of each dose relative to the historical control and determine the optimal dose through between‐dose comparisons. The triple‐outcome decision rule accommodates a reasonable range of inconclusive trial outcomes, allowing multiple factors to be considered when efficacy data alone are insufficient to identify the optimal dose. Additionally, the Bayesian dynamic linear model facilitates association and adaptive information borrowing across adjacent doses. A systematic calibration procedure is also proposed to optimize the TODO design under various scenarios. These innovative features enhance the performance and flexibility of the decision‐making process. The desirable operating characteristics of the TODO design, demonstrated through simulation studies, are evident in scenarios using a primary efficacy outcome as well as those with coprimary toxicity and efficacy outcomes.

While the triple‐outcome approach enhances flexibility in trial decision‐making, it also introduces added complexity in trial planning, especially when more than two doses are involved. Investigators should carefully weigh the advantages of the triple‐outcome decision rule—such as increased flexibility and the potential for more informed dose selection—against the challenges it may pose. These challenges include the need for more sophisticated statistical analyses, potential added uncertainty in decision‐making, and possibly longer timelines for decisions. If this added complexity becomes a significant obstacle, the proposed design can be simplified to a conventional dichotomous decision framework by setting $c_{1}=c_{2}$.

The TODO framework is general and can be further generalized in various aspects. Following the widely adopted Simon's two‐stage design [36], we employ a two‐stage format in the proposed design, primarily to simplify trial conduct and design calibration. However, the design can be extended to a multistage approach with more frequent per‐dose futility or toxicity monitoring. Although most trials compare only two doses, some may investigate more than two doses. In the latter scenarios, we assume a monotonic dose–efficacy relationship to simplify the decision rule (which is not required for two‐dose trials). While this approach offers simplicity, it might not fully capture the complexity of dose–efficacy patterns observed in real‐world situations. Therefore, developing more flexible statistical models and decision rules is essential to accommodate a wider range of dose–efficacy relationships. It is also important to recognize that dose optimization should consider multiple factors. Incorporating additional information such as pharmacokinetics, pharmacodynamics, and patient‐reported outcomes into the TODO framework could lead to a more comprehensive evaluation of the doses. R codes for optimizing design parameters and conducting simulation studies for the TODO design are available at the GitHub repository https://github.com/ruitaolin/TODO.

## Conflicts of Interest

The authors declare no conflicts of interest.

## Supporting information


Data S1.

## Data Availability

Data sharing is not applicable to this article as no datasets were generated or analyzed during the current study.
