# Supplementary material for: TODO: A Triple‐Outcome Double‐Criterion Optimal Design for Dose Monitoring‐and‐Optimization in Multi‐Dose Randomized Trials
Source: Stat Med. 2025 May 19;44(10-12):e70090. doi: 10.1002/sim.70090 (PMC12089520; doi:10.1002/sim.70090)
Supplement: Supplementary file 1 — Data S1. [file SIM-44-0-s001.pdf]

# Supplementary Materials for "TODO: A Triple-Outcome Double-Criterion Optimal Design for Dose Testing-and-Optimization in Multi-Dose Randomized Trials"

**Jingyi Zhang[1], Heng Zhou[2], Nolan A. Wages[3], Zifang Guo[2], Fang Liu[2], Thomas Jemielita[2], Fangrong Yan[1], and Ruitao Lin[4*]**

# S1 Additional simulation results

## S1.1 Calibrated parameter values

Tables S1–S3 summarize the calibrated values for the design parameters used in the TODO, TODO-BB, and DREAMM-2 designs, respectively.

## S1.2 Sensitivity to the hyperparameters

Figure S6 illustrates the design's performance across various values of the hyperparameter $\tau^2$ and $\theta$ in scenarios 1.1–1.9, demonstrating the design's robustness to variations in these two parameters.

## S1.3 Larger sample sizes

We have evaluated the performance of the proposed design with larger sample sizes of 36 and 91 per arm under Scenarios 1.1–1.9, keeping other configurations consistent with those in Section 4.1. As shown in Table S1, a sample size of 36 corresponds to a FWER of 5% and an OMP of 95.0%. Similarly, a sample size of 91 corresponds to a FWER of 1% and an OMP of 99.8% (e.g., a per-dose power of the TODO design $\geq$ 95%). Simulation results for the three competing designs are presented in Table S5 and Table S6. The average bias and MSE of efficacy estimates for the TODO and TODO-BB designs are summarized in Figure

S7 and Figure S8. Additionally, the impact of $w_l$ on design performance based on these two larger sample sizes is illustrated in Figure S4 and Figure S5.

Table S4 presents the simulation results for the generalized TODO design under Scenarios 2.1–2.15 with a sample size of 32, keeping other configurations consistent with those in Section 5.2. As shown in Table S1, this sample size of 32 corresponds to a FWER of 5% and an OMP of 95.0%.

Overall, the proposed TODO design maintains its advantages and performs even better with the increased sample size.

### S1.4 Different values of $p_0$ and $p_A$

We have considered different scenarios for $p_0$ and $p_A$, which reflect various historical control rates and alternative rates deemed promising, respectively. The configurations of the competing designs and other trial settings remain unchanged. All competing designs have undergone the same calibration procedure as outlined in Section 4.1. The resulting design parameters are provided in Table S1. The simulation results with $(p_0, p_A) = (0.10, 0.30)$ are present in Table S7, and those with $(p_0, p_A) = (0.3, 0.5)$ are reported in Table S8. Generally, the findings align with those in Section 4.2: the TODO design consistently achieves a desirable correct selection rate across all scenarios and typically results in the lowest weighted loss.

## S2 Comparing more than two doses

### S2.1 Design details

The TODO design for multi-dose trials is largely similar to the two-dose trials described in the main paper. However, there are some unique elements incorporated to simplify decision-making and design calibration for multi-dose trials. The optimal dose is similarly defined as the lowest dose that exhibits promising efficacy and is non-inferior to the highest dose, i.e.,

$p_j \geq p_0$ and $p_J - p_j \leq \delta_1$.

Similar to the two-dose case, we use $\Pr(p_j > p_0|\mathcal{D}_k)$ for per-dose monitoring and $\text{PP}_{\text{NI}}(j) = \Pr(p_J - p_j < \delta_1 \mid \mathcal{D}_2)$ for between-dose comparison. In the final analysis when more than two doses pass the per-dose monitoring, the between-dose comparison procedure for final dose selection becomes more complex. Specifically, each pairwise comparison, based on $\text{PP}_{\text{NI}}(j) = \Pr(p_J - p_j < \delta_1|\mathcal{D}_2)$, can yield three possible outcomes: inferiority of $d_j$ if $\text{PP}_{\text{NI}}(j) \leq c_1$, non-inferiority of $d_j$ if $\text{PP}_{\text{NI}}(j) > c_2$, and inconclusiveness if $c_1 < \text{PP}_{\text{NI}}(j) \leq c_2$, for $j = 1, \ldots, J-1$. This results in a total of $3^{J-1}$ possible combinations when comparing $J-1$ lower doses with the highest dose $J$. For example, when there are three active doses, there are 9 possible trial outcomes as shown in Table S9. In addition, non-meaningful results may arise in arises in multi-dose comparisons with $J > 2$ active doses. For instance, in a trial with three doses, dose $d_1$ may be non-inferior to $d_3$, but $d_2$ is inferior to $d_3$. Here, even if $d_1$ is selected as the optimal dose, the inferiority of $d_2$ poses interpretative challenges for the final dose selection.

To address this challenging issue, we further impose a constraint of a non-decreasing dose-efficacy relationship, which is a reasonable assumption in most real-world scenarios. By imposing this constraint, the number of possible trial outcomes is reduced to $J(J-1)/2$. This reduction is significant because the monotonic nature of the dose-efficacy relationship ensures that non-meaningful results are eliminated, enhancing the trial's interpretative clarity. For example, with $J = 3$ doses, Table S9 displays the six possible trial outcomes.

To impose the non-decreasing relationship, we apply the isotonic regression (Bril et al., 1984) to $\Pr(p_j > p_0|\mathcal{D}_k), j = 1, \ldots, J,\ k = 1, 2$ and $\text{PP}_{\text{NI}}(j), j = 1, \ldots, J-1$, respectively. This ensures that if any dose $j$ is found to be futile (or inferior to the highest dose), all of its lower doses must also be futile (or inferior to the highest dose), thereby maintaining a logical and consistent evaluation of dose efficacy.

## S2.2 Parameter optimization

We extend the two-step calibration procedure to optimize the sample sizes $n$ and $m_1$, as well as the decision cutoffs $a_1$, $a_2$, $c_1$, and $c_2$, for the TODO design in trials involving more than two doses. In Step 1, the global null is defined as $\cap_{j=1}^{J} H_{01}(j)$ and the global alternative is defined as $\cap_{j=1}^{J} H_{A1}(j)$. In Step 2, to identify the optimal values of $c_1$ and $c_2$, we consider two special sets of alternative hypotheses: $H_A^*(j) : (p_1, \ldots, p_j, p_{j+1}, \ldots, p_J) = (p_A, \ldots, p_A, p_A + \delta_1, \ldots, p_A + \delta_1)$ with the lower $j$ doses being all non-inferior to the higher $J - j$ doses; and $H_A^+(j) : (p_1, \ldots, p_j, p_{j+1}, \ldots, p_J) = (p_A, \ldots, p_A, p_A + \delta_2, \ldots, p_A + \delta_2)$ with the lower $j$ doses being all inferior to the higher $J - j$ doses, where $j = 1, \ldots, J - 1$. Under $H_A^*(j)$ and $H_A^+(j)$, $j = 1, \ldots, J - 1$, the three key performance metrics are defined as:

(a) The incorrect decision rate (IDR), defined as the average probability of making any incorrect trial decisions under the alternative hypotheses, i.e.,

$$\text{IDR} = \sum_{j=1}^{J-1} \{\Pr(d_1 \text{ is not selected} \mid H_A^*(j)) + \Pr(d_{j+1} \text{ is not selected} \mid H_A^+(j))\}/\{2(J-1)\}.$$

(b) The size of the inconclusive region size (SIR), defined as the probability of having an inconclusive trial outcome averaged across $H_A^*(j)$ and $H_A^+(j)$, $j = 1, \ldots, J - 1$.

(c) The maximum rate of selecting an inadequate dose (MRID), defined as the maximum probability of incorrectly selecting a lower inferior dose across $H_A^+(j)$, $j = 1 \ldots, J - 1$, i.e., $\max_{1 \le j \le J-1} \{\Pr(\text{Select } d_{j'} \le d_j \mid H_A^+(j)\}$.

In Algorithm S1, we detail the steps for calibrating the design parameters for multi-dose trials. Although the TODO design is versatile and efficient for multi-dose trials, careful and cautious planning is essential when considering trials with more than two doses. The increase in the number of candidate doses leads to a higher number of between-dose comparison pairs, which in turn can result in a need for larger sample sizes. This is due to the increased variability arising from multiple pairwise dose comparisons. Therefore, we caution that studies involving a greater number of doses may introduce additional complexities. Prior

to conducting such trials, selecting the most appropriate candidate doses is crucial. This selection should be informed by multiple factors, including phase I trial results and expert opinions. The specifics of selecting candidate doses, however, fall outside the scope of this paper.

### S2.3 Simulation studies with three doses

We investigate the performance of the TODO design in a three-dose comparison using additional simulation studies. With three doses, the likelihood of an inconclusive result increases. To mitigate this, we adopt a larger value of $w_l = 0.6$ to reduce the SIR, while keeping other design parameters the same as those specified for a two-dose comparison (see Section 4.1). The maximum sample size per dose arm is 22 patients, and an interim analysis for futility monitoring is conducted after enrolling 10 patients per arm to minimize the average sample size under the null scenario. In this section, the DREAMM-2 design is not included because its decision rule is not applicable for more than two doses. Table S10 summarizes the simulation results under nine different scenarios, clearly demonstrating that the TODO design maintains robust performance even in this more complex three-dose trial setting. Figure S9 illustrates the average bias and mean square error (MSE) across these scenarios, further highlighting the superiority of the TODO design's superior accuracy and stability in estimation. Figure S10 illustrates the influence of $w_l$ on the cutoff values and the selection percentage of the optimal dose.

## References

Gordon Bril, Richard Dykstra, Carolyn Pillers, and Tim Robertson. Algorithm as 206: Isotonic regression in two independent variables. *Journal of the Royal Statistical Society. Series C (Applied Statistics)*, 33(3):352–357, 1984. ISSN 00359254, 14679876. URL http://www.jstor.org/stable/2347723.

**Algorithm S1** Parameter optimization algorithm for $(n, m_1, a_1, a_2, c_1, c_2)$

**Step 1:** Optimize $(n, m_1, a_1, a_2)$.

1. Specify the historical control rate $p_0$, the promising efficacy rate $p_A > p_0$, the search range $\mathcal{A}$ for $(\lambda, a_2)$ (with $a_1 = a_2(m_1/n)^\lambda$), the search range $\mathcal{M}$ for $m_1$, the nominal level $\alpha_1$ for Pr(Identify a dose as effective | $p_1 = \cdots = p_J = p_0$), and the target overall monitoring power $\beta_1$ for Pr(Identify a dose as effective | $p_1 = \cdots = p_J = p_A$). In this paper, we set $\mathcal{A} = [\log(1)/\log(0.5), \log(0.9975)/\log(0.5), \ldots, \log(0.5025)/\log(0.5), \log(0.5)/\log(0.5)] \times [0.5, 0.5025, \ldots, 0.995, 0.9975]$ and $\mathcal{M} = [1/3n, 2/3n]$.

2. Given a reasonably small value $n$ (e.g., $n = 15$), conduct simulation studies to collect all pairs of $(m_1, a_1, a_2)$ among $\mathcal{M} \cap \mathcal{A}$, denoted as $\mathcal{M}_1 \cap \mathcal{A}_1$, that satisfies the constraint of Pr(Identify a dose as effective | $p_1 = \cdots = p_J = p_0) \leq \alpha_1$

3. For each unique $m_1$ in $\mathcal{M}_1$, identify the optimal pair $(a_1^*, a_2^*)$ among $\mathcal{A}_1$ that maximizes the overall monitoring power Pr(Identify a dose as effective | $p_1 = \cdots = p_J = p_A$), and denote the corresponding overall monitoring power as OMP($m_1$). Denote the maximum overall monitoring power among $\mathcal{M}_1$ as $\text{OMP}^* = \max_{m_1 \in \mathcal{M}_1} \{\text{OMP}(m_1)\}$.

4. Within the range of $m_1$ values that satisfy $\text{OMP}(m_1) \geq (\text{OMP}^* - 1\%)$, identify the optimal combination of $(m_1^*, a_1^*, a_2^*)$ that minimizes the average sample size under the global null hypothesis $p_1 = \cdots = p_J = p_0$.

5. If $\text{OMP}(m_1^*)$ is less than the target overall monitoring power $\beta_1$, increase the per-dose total sample size $n$ by one unit. Otherwise, stop Step 1, and the optimal set $(n^*, m_1^*, \alpha_1^*, \alpha_2^*)$ is identified.

**Step 2:** Optimize $(c_1, c_2)$.

1. Specify the non-inferiority margin $\delta_1$, the inferiority margin $\delta_2 > \delta_1$, the weight $w_l$, the search range $\mathcal{C}$ for $(c_1, c_2)$, the upper limit $\alpha_2$ for MRID, the upper limit $\alpha_3$ for SIR. In this paper, we set $\mathcal{C} = [0.01, 0.02, \ldots, 0.98, 0.99] \times [0.01, 0.02, \ldots, 0.98, 0.99]$ with $c_1 \leq c_2$.

2. Based on $p_0$, $p_A$, $\delta_1$, $\delta_2$, define the two alternative sets as

$$H_A^*(j) : (p_1, \ldots, p_j, p_{j+1}, \ldots, p_J) = (p_A, \ldots, p_A, p_A + \delta_1, \ldots, p_A + \delta_1),$$

$$H_A^+(j) : (p_1, \ldots, p_j, p_{j+1}, \ldots, p_J) = (p_A, \ldots, p_A, p_A + \delta_2, \ldots, p_A + \delta_2),$$

for $j = 1, \ldots, J - 1$.

3. Based on $(n^*, m_1^*, a_1^*, a_2^*)$, conduct simulation studies to collect all pairs of $(c_1, c_2)$ among $\mathcal{C}$, denoted as $\mathcal{C}_1$, that satisfies the constraints of MRID and SIR.

4. Identify the optimal pair $(c_1^*, c_2^*)$ among $\mathcal{C}_1$ that minimizes the average value of WL = IDR + $w_l$SIR across the two alternative sets.

Table S1: Summary of the family-wise type I error rate (FWER), overall monitoring power (OMP), and the corresponding optimal parameters $(m_1^*, n^*, a_1^*, a_2^*, c_1^*, c_2^*)$ for the TODO design under different simulation settings. In all settings, the size of the inconclusive region (SIR) and the maximum rate of selecting an inadequate dose (MRID) are controlled at $\alpha_2 = 20\%$ and $\alpha_3 = 15\%$, with the discount factor $w_l = 0.40$.

| Setting | FWER | OMP | Null | Alternative | $n^*$ | $m_1^*$ | $a_1^*$ | $a_2^*$ | $c_1^*$ | $c_2^*$ |
|---|---|---|---|---|---|---|---|---|---|---|
| Scenarios 1.1–1.9 | 10% | 95.0% | $p_0 = 0.2$ | $p_A = 0.4$ | 29 | 10 | 0.3155 | 0.9150 | 0.53 | 0.61 |
| Scenarios 1.1–1.9 | 5% | 95.0% | $p_0 = 0.2$ | $p_A = 0.4$ | 36 | 15 | 0.3990 | 0.9575 | 0.48 | 0.57 |
| Scenarios 1.1–1.9 | 1% | 99.8% | $p_0 = 0.2$ | $p_A = 0.4$ | 91 | 45 | 0.5892 | 0.9900 | 0.24 | 0.34 |
| Scenarios 2.1–2.9 | 10% | 95.0% | $p_0 = 0.2$, $q_0 = 0.4$ | $p_A = 0.4$, $q_A = 0.25$ | 26 | 11 | 0.3057 | 0.7225 | 0.48 | 0.62 |
| Scenarios 2.1–2.9 | 5% | 95.0% | $p_0 = 0.2$, $q_0 = 0.4$ | $p_A = 0.4$, $q_A = 0.25$ | 32 | 13 | 0.3474 | 0.8025 | 0.43 | 0.58 |
| Scenarios 3.1–3.9 | 10% | 95.0% | $p_0 = 0.1$ | $p_A = 0.3$ | 20 | 12 | 0.5205 | 0.8675 | 0.56 | 0.65 |
| Scenarios 4.1–4.9 | 10% | 95.0% | $p_0 = 0.3$ | $p_A = 0.5$ | 31 | 13 | 0.4101 | 0.9200 | 0.46 | 0.55 |
| Scenarios 5.1–5.9 | 10% | 95.0% | $p_0 = 0.2$ | $p_A = 0.4$ | 22 | 10 | 0.4636 | 0.8700 | 0.45 | 0.53 |

Table S2: Summary of target family-wise type I error rate (FWER), and the corresponding optimal parameters $(n^*, m_1^*, a_1^*, a_2^*, c_1^*, c_2^*)$ for the TODO-BB design under different simulation settings.

| Setting | FWER | Null | Alternative | $n^*$ | $m_1^*$ | $a_1^*$ | $a_2^*$ | $c_1^*$ | $c_2^*$ |
|---|---|---|---|---|---|---|---|---|---|
| Scenarios 1.1–1.9 | 10% | $p_0 = 0.2$ | $p_A = 0.4$ | 29 | 10 | 0.4369 | 0.9575 | 0.32 | 0.45 |
| Scenarios 1.1–1.9 | 5% | $p_0 = 0.2$ | $p_A = 0.4$ | 36 | 15 | 0.4354 | 0.9825 | 0.28 | 0.42 |
| Scenarios 1.1–1.9 | 1% | $p_0 = 0.2$ | $p_A = 0.4$ | 91 | 45 | 0.6157 | 0.9925 | 0.12 | 0.19 |
| Scenarios 3.1–3.9 | 10% | $p_0 = 0.1$ | $p_A = 0.3$ | 20 | 12 | 0.4045 | 0.8900 | 0.48 | 0.53 |
| Scenarios 4.1–4.9 | 10% | $p_0 = 0.3$ | $p_A = 0.5$ | 31 | 13 | 0.6900 | 0.8975 | 0.34 | 0.50 |
| Scenarios 5.1–5.9 | 10% | $p_0 = 0.2$ | $p_A = 0.4$ | 22 | 10 | 0.4897 | 0.9800 | 0.35 | 0.47 |

Table S3: Summary of target family-wise type I error rate (FWER), sample sizes $(m_1, n)$, stopping boundaries for futility at interim analysis (IA) and final analysis (FA), and the cutoff value $c'$ for the DREAMM-2 design under different simulation settings.

| Setting | FWER | Null | Alternative | $n^*$ | $m_1^*$ | Stop if # response <= | | $c'$ |
|---|---|---|---|---|---|---|---|---|
| | | | | | | IA | FA | |
| Scenarios 1.1–1.9 | 10% | $p_0 = 0.2$ | $p_A = 0.4$ | 29 | 10 | 1 | 9 | 0.6 |
| Scenarios 1.1–1.9 | 5% | $p_0 = 0.2$ | $p_A = 0.4$ | 36 | 15 | 2 | 12 | 0.6 |
| Scenarios 1.1–1.9 | 1% | $p_0 = 0.2$ | $p_A = 0.4$ | 91 | 45 | 12 | 28 | 0.6 |
| Scenarios 3.1–3.9 | 10% | $p_0 = 0.1$ | $p_A = 0.3$ | 20 | 12 | 1 | 4 | 0.6 |
| Scenarios 4.1–4.9 | 10% | $p_0 = 0.3$ | $p_A = 0.5$ | 31 | 13 | 3 | 14 | 0.6 |

Table S4: Simulation results for simultaneously evaluating toxicity and efficacy with a sample size of 32 per dose. Optimal doses and correct selection decisions are highlighted in boldface. Selection %: Percentage of selecting the dose in the final analysis. SIR: Percentage of having an inconclusive result. IDR: Rate of incorrect decisions. WL: Weighted loss (with $w_l = 0.40$). ASS: Average sample size. Go %: Percentage of making a "go" decision in the futility monitoring, i.e., the per-dose power. FWER/OMP %: Percentage of trials identifying at least one dose as effective in the futility monitoring, which represents the family-wise type I error rate (FWER) in Scenario 2.1 and the overall monitoring power (OMP) in the other scenarios.

| Scenario | Selection % | | SIR | IDR | WL | ASS | | Go % | | FWER/OMP % |
|---|---|---|---|---|---|---|---|---|---|---|
| | $d_1$ | $d_2$ | | | | $d_1$ | $d_2$ | $d_1$ | $d_2$ | |
| 2.1 | 2.6 | 2.4 | 0.0 | 5.0 | 5.0 | 21.2 | 21.3 | 2.6 | 2.8 | 5.0 |
| 2.2 | **61.8** | 21.5 | 12.0 | 26.3 | 31.1 | 31.2 | 31.2 | 85.7 | 85.3 | 95.2 |
| 2.3 | 19.8 | **64.6** | 11.8 | 23.6 | 28.3 | 31.2 | 31.5 | 85.9 | 89.0 | 96.2 |
| 2.4 | **51.1** | 30.2 | 14.5 | 34.4 | 40.2 | 31.2 | 31.3 | 86.0 | 87.7 | 95.8 |
| 2.5 | 9.2 | 4.3 | 0.3 | 13.7 | 13.8 | 23.9 | 23.2 | 9.6 | 6.6 | 13.7 |
| 2.6 | 5.9 | 1.2 | 0.1 | 7.2 | 7.2 | 23.0 | 20.0 | 6.1 | 1.5 | 7.2 |
| 2.7 | 11.7 | **71.4** | 6.0 | 22.6 | 25.0 | 27.3 | 31.2 | 27.9 | 88.1 | 89.1 |
| 2.8 | 13.6 | **74.8** | 11.4 | 13.8 | 18.4 | 31.7 | 31.9 | 96.3 | 97.8 | 99.8 |
| 2.9 | 13.5 | **75.8** | 10.6 | 13.6 | 17.8 | 31.9 | 31.9 | 99.4 | 97.8 | 99.9 |
| 2.10 | **69.7** | 16.5 | 5.2 | 25.1 | 27.2 | 31.4 | 27.6 | 90.1 | 30.3 | 91.5 |
| 2.11 | **63.3** | 21.6 | 14.7 | 22.0 | 27.9 | 31.7 | 31.7 | 96.4 | 96.6 | 99.6 |
| 2.12 | **68.7** | 19.3 | 11.8 | 19.5 | 24.2 | 31.8 | 31.8 | 96.8 | 97.1 | 99.8 |
| 2.13 | **68.7** | 18.4 | 12.9 | 18.4 | 23.6 | 32.0 | 31.9 | 99.8 | 97.5 | 100.0 |
| 2.14 | **91.1** | 0.3 | 0.4 | 8.5 | 8.6 | 31.6 | 25.0 | 91.8 | 13.8 | 91.8 |
| 2.15 | **84.9** | 6.3 | 8.7 | 6.4 | 9.9 | 31.9 | 31.7 | 99.5 | 96.7 | 99.9 |

Table S5: Simulation results for the two-dose trials with a sample size of 36 per dose. Optimal doses and correct selection decisions are highlighted in boldface. Selection %: Percentage of selecting the dose in the final analysis. SIR: Percentage of having an inconclusive result. IDR: Rate of incorrect decisions. WL: Weighted loss (with $w_l = 0.40$). ASS: Average sample size. Go %: Percentage of making a "go" decision in the futility monitoring, i.e., the per-dose power. FWER/OMP %: Percentage of trials identifying at least one dose as effective in the futility monitoring, which represents the family-wise type I error rate (FWER) in Scenario 1.1 and the overall monitoring power (OMP) in the other scenarios.

| Method | Selection % | | SIR | IDR | WL | ASS | | Go % | | FWER/OMP % |
|---|---|---|---|---|---|---|---|---|---|---|
| | $d_1$ | $d_2$ | | | | $d_1$ | $d_2$ | $d_1$ | $d_2$ | |
| Scenario 1.1: $(p_1, p_2) = (0.20, 0.20)$ | | | | | | | | | | |
| TODO | 2.1 | 2.2 | 0.0 | 4.3 | 4.3 | 23.1 | 25.7 | 2.1 | 2.7 | 4.3 |
| TODO-BB | 2.0 | 1.8 | 0.0 | 3.8 | 3.8 | 27.5 | 27.8 | 2.0 | 1.8 | 3.8 |
| DREAMM-2 | 1.7 | 1.7 | 0.0 | 3.5 | 3.5 | 27.5 | 27.5 | 1.7 | 1.8 | 3.5 |
| Scenario 1.2: $(p_1, p_2) = (\mathbf{0.40}, 0.40)$ | | | | | | | | | | |
| TODO | **72.6** | 16.2 | 6.7 | 20.7 | 23.4 | 35.3 | 35.6 | 87.6 | 88.3 | 95.5 |
| TODO-BB | **61.8** | 24.1 | 7.2 | 31.0 | 33.9 | 35.4 | 35.4 | 73.0 | 74.1 | 93.1 |
| DREAMM-2 | **56.3** | 36.5 | 0.0 | 43.7 | 43.7 | 35.5 | 35.4 | 73.5 | 73.5 | 92.8 |
| Scenario 1.3: $(p_1, p_2) = (\mathbf{0.40}, 0.45)$ | | | | | | | | | | |
| TODO | **60.6** | 27.7 | 10.2 | 29.2 | 33.3 | 35.6 | 35.9 | 89.4 | 96.3 | 98.5 |
| TODO-BB | **51.2** | 34.8 | 11.4 | 37.4 | 42.0 | 35.4 | 35.8 | 73.0 | 89.7 | 97.3 |
| DREAMM-2 | **44.1** | 52.9 | 0.0 | 55.9 | 55.9 | 35.4 | 35.8 | 73.2 | 88.7 | 97.0 |
| Scenario 1.4: $(p_1, p_2) = (0.40, \mathbf{0.60})$ | | | | | | | | | | |
| TODO | 15.6 | **75.0** | 9.4 | 15.6 | 19.4 | 35.6 | 36.0 | 89.4 | 100.0 | 100.0 |
| TODO-BB | 13.4 | **73.9** | 12.6 | 13.5 | 18.5 | 35.4 | 36.0 | 73.0 | 99.8 | 100.0 |
| DREAMM-2 | 8.3 | **91.7** | 0.0 | 8.3 | 8.3 | 35.4 | 36.0 | 73.8 | 99.8 | 99.9 |
| Scenario 1.5: $(p_1, p_2) = (0.20, \mathbf{0.40})$ | | | | | | | | | | |
| TODO | 3.9 | **71.4** | 1.1 | 27.5 | 27.9 | 29.8 | 34.9 | 7.4 | 76.1 | 76.4 |
| TODO-BB | 1.3 | **72.9** | 0.4 | 26.7 | 26.9 | 27.5 | 35.4 | 2.0 | 74.1 | 74.6 |
| DREAMM-2 | 1.1 | **72.4** | 0.0 | 27.6 | 27.6 | 27.5 | 35.4 | 2.0 | 73.1 | 73.5 |
| Scenario 1.6: $(p_1, p_2) = (0.40, \mathbf{0.70})$ | | | | | | | | | | |
| TODO | 2.6 | **94.1** | 3.4 | 2.6 | 3.9 | 35.5 | 36.0 | 86.7 | 100.0 | 100.0 |
| TODO-BB | 2.1 | **93.8** | 4.1 | 2.1 | 3.8 | 35.4 | 36.0 | 73.0 | 100.0 | 100.0 |
| DREAMM-2 | 1.3 | **98.7** | 0.0 | 1.3 | 1.3 | 35.5 | 36.0 | 73.8 | 100.0 | 100.0 |
| Scenario 1.7: $(p_1, p_2) = (\mathbf{0.40}, 0.43)$ | | | | | | | | | | |
| TODO | **66.0** | 22.7 | 8.8 | 25.2 | 28.7 | 35.5 | 35.8 | 88.8 | 93.9 | 97.5 |
| TODO-BB | **56.0** | 30.4 | 9.6 | 34.3 | 38.2 | 35.4 | 35.6 | 73.0 | 84.8 | 96.1 |
| DREAMM-2 | **50.0** | 45.9 | 0.0 | 50.1 | 50.1 | 35.4 | 35.6 | 74.1 | 83.5 | 95.9 |
| Scenario 1.8: $(p_1, p_2) = (\mathbf{0.45}, 0.40)$ | | | | | | | | | | |
| TODO | **85.2** | 7.4 | 4.8 | 10.0 | 11.9 | 35.6 | 35.7 | 95.1 | 89.3 | 97.4 |
| TODO-BB | **79.3** | 11.6 | 6.3 | 14.4 | 16.9 | 35.8 | 35.4 | 88.9 | 74.1 | 97.1 |
| DREAMM-2 | **74.2** | 22.8 | 0.0 | 25.9 | 25.9 | 35.7 | 35.4 | 88.2 | 74.0 | 97.0 |
| Scenario 1.9: $(p_1, p_2) = (\mathbf{0.60}, 0.40)$ | | | | | | | | | | |
| TODO | **97.3** | 0.2 | 0.6 | 2.1 | 2.3 | 35.6 | 35.6 | 98.1 | 89.2 | 98.1 |
| TODO-BB | **98.8** | 0.4 | 0.8 | 0.4 | 0.7 | 36.0 | 35.4 | 99.9 | 74.1 | 100.0 |
| DREAMM-2 | **98.1** | 1.8 | 0.0 | 1.9 | 1.9 | 36.0 | 35.5 | 99.8 | 73.2 | 99.9 |

Table S6: Simulation results for the two-dose trials with a sample size of 91 per dose. Optimal doses and correct selection decisions are highlighted in boldface. Selection %: Percentage of selecting the dose in the final analysis. SIR: Percentage of having an inconclusive result. IDR: Rate of incorrect decisions. WL: Weighted loss (with $w_l = 0.40$). ASS: Average sample size. Go %: Percentage of making a "go" decision in the futility monitoring, i.e., the per-dose power. FWER/OMP %: Percentage of trials identifying at least one dose as effective in the futility monitoring, which represents the family-wise type I error rate (FWER) in Scenario 1.1 and the overall monitoring power (OMP) in the other scenarios.

| Method | Selection % | | SIR | IDR | WL | ASS | | Go % | | FWER/OMP % |
|---|---|---|---|---|---|---|---|---|---|---|
| | $d_1$ | $d_2$ | | | | $d_1$ | $d_2$ | $d_1$ | $d_2$ | |
| Scenario 1.1: $(p_1, p_2) = (0.20, 0.20)$ | | | | | | | | | | |
| TODO | 0.5 | 0.6 | 0.0 | 1.1 | 1.1 | 55.1 | 62.1 | 0.5 | 0.6 | 1.1 |
| TODO-BB | 0.4 | 0.5 | 0.0 | 0.9 | 0.9 | 64.0 | 64.3 | 0.4 | 0.5 | 0.9 |
| DREAMM-2 | 0.4 | 0.4 | 0.0 | 0.8 | 0.8 | 49.5 | 49.5 | 0.4 | 0.4 | 0.8 |
| Scenario 1.2: $(p_1, p_2) = (\mathbf{0.40}, 0.40)$ | | | | | | | | | | |
| TODO | **93.3** | 4.0 | 2.5 | 4.2 | 5.2 | 90.8 | 90.9 | 98.1 | 98.5 | 99.8 |
| TODO-BB | **91.5** | 6.3 | 2.0 | 6.6 | 7.4 | 90.8 | 90.8 | 95.1 | 95.3 | 99.8 |
| DREAMM-2 | **58.3** | 41.2 | 0.0 | 41.7 | 41.7 | 89.2 | 89.1 | 93.2 | 93.2 | 99.5 |
| Scenario 1.3: $(p_1, p_2) = (\mathbf{0.40}, 0.45)$ | | | | | | | | | | |
| TODO | **80.9** | 12.3 | 6.8 | 12.3 | 15.0 | 90.9 | 91.0 | 98.2 | 99.9 | 100.0 |
| TODO-BB | **80.1** | 13.6 | 6.3 | 13.6 | 16.2 | 90.8 | 91.0 | 95.1 | 99.6 | 100.0 |
| DREAMM-2 | **31.7** | 68.2 | 0.0 | 68.3 | 68.3 | 88.9 | 90.7 | 92.7 | 99.1 | 99.9 |
| Scenario 1.4: $(p_1, p_2) = (0.40, \mathbf{0.60})$ | | | | | | | | | | |
| TODO | 11.8 | **80.6** | 7.6 | 11.8 | 14.8 | 90.9 | 91.0 | 98.0 | 100.0 | 100.0 |
| TODO-BB | 11.9 | **80.8** | 7.3 | 11.9 | 14.8 | 90.8 | 91.0 | 95.1 | 100.0 | 100.0 |
| DREAMM-2 | 0.6 | **99.4** | 0.0 | 0.6 | 0.6 | 89.0 | 91.0 | 93.0 | 100.0 | 100.0 |
| Scenario 1.5: $(p_1, p_2) = (0.20, \mathbf{0.40})$ | | | | | | | | | | |
| TODO | 1.2 | **94.5** | 0.3 | 5.2 | 5.3 | 70.1 | 90.8 | 1.8 | 96.0 | 96.0 |
| TODO-BB | 0.4 | **94.9** | 0.1 | 5.1 | 5.1 | 64.0 | 90.8 | 0.4 | 95.3 | 95.3 |
| DREAMM-2 | 0.0 | **92.4** | 0.0 | 7.6 | 7.6 | 49.7 | 88.8 | 0.4 | 92.4 | 92.4 |
| Scenario 1.6: $(p_1, p_2) = (0.40, \mathbf{0.70})$ | | | | | | | | | | |
| TODO | 0.5 | **98.9** | 0.6 | 0.5 | 0.8 | 90.8 | 91.0 | 97.6 | 100.0 | 100.0 |
| TODO-BB | 0.5 | **98.8** | 0.7 | 0.5 | 0.7 | 90.8 | 91.0 | 95.1 | 100.0 | 100.0 |
| DREAMM-2 | 0.0 | **100.0** | 0.0 | 0.0 | 0.0 | 88.8 | 91.0 | 92.8 | 100.0 | 100.0 |
| Scenario 1.7: $(p_1, p_2) = (\mathbf{0.40}, 0.43)$ | | | | | | | | | | |
| TODO | **87.2** | 8.0 | 4.7 | 8.1 | 10.0 | 90.9 | 91.0 | 98.2 | 99.7 | 100.0 |
| TODO-BB | **85.8** | 9.8 | 4.4 | 9.8 | 11.6 | 90.8 | 90.9 | 95.1 | 98.8 | 100.0 |
| DREAMM-2 | **42.1** | 57.7 | 0.0 | 57.9 | 57.9 | 89.0 | 90.2 | 92.8 | 97.6 | 99.9 |
| Scenario 1.8: $(p_1, p_2) = (\mathbf{0.45}, 0.40)$ | | | | | | | | | | |
| TODO | **98.6** | 0.6 | 0.7 | 0.7 | 1.0 | 90.9 | 90.9 | 99.8 | 98.5 | 99.8 |
| TODO-BB | **98.4** | 1.0 | 0.7 | 1.0 | 1.2 | 91.0 | 90.8 | 99.6 | 95.3 | 100.0 |
| DREAMM-2 | **81.5** | 18.5 | 0.0 | 18.6 | 18.6 | 90.6 | 89.0 | 98.8 | 92.9 | 100.0 |
| Scenario 1.9: $(p_1, p_2) = (\mathbf{0.60}, 0.40)$ | | | | | | | | | | |
| TODO | **99.7** | 0.0 | 0.0 | 0.3 | 0.3 | 90.9 | 90.9 | 99.7 | 98.4 | 99.7 |
| TODO-BB | **100.0** | 0.0 | 0.0 | 0.0 | 0.0 | 91.0 | 90.8 | 100.0 | 95.3 | 100.0 |
| DREAMM-2 | **99.8** | 0.2 | 0.0 | 0.2 | 0.2 | 91.0 | 89.0 | 100.0 | 93.0 | 100.0 |

Table S7: Simulation results for the two-dose trials with $p_0 = 0.1$ and $p_1 = 0.3$. Optimal doses and correct selection decisions are highlighted in boldface. Selection %: Percentage of selecting the dose in the final analysis. SIR: Percentage of having an inconclusive result. IDR: Rate of incorrect decisions. WL: Weighted loss (with $w_l = 0.40$). ASS: Average sample size. Go %: Percentage of making a “go” decision in the futility monitoring, i.e., the per-dose power. FWER/OMP %: Percentage of trials identifying at least one dose as effective in the futility monitoring, which represents the family-wise type I error rate (FWER) in Scenario 3.1 and the overall monitoring power (OMP) in the other scenarios.

| Method | Selection % | | SIR | IDR | WL | ASS | | Go % | | FWER/OMP % |
|---|---|---|---|---|---|---|---|---|---|---|
| | $d_1$ | $d_2$ | | | | $d_1$ | $d_2$ | $d_1$ | $d_2$ | |
| Scenario 3.1: $(p_1, p_2) = (0.10, 0.10)$ | | | | | | | | | | |
| TODO | 4.7 | 4.4 | 0.1 | 9.2 | 9.2 | 13.5 | 15.0 | 5.0 | 6.0 | 9.2 |
| TODO-BB | 4.1 | 4.2 | 0.0 | 8.3 | 8.3 | 14.7 | 14.7 | 4.1 | 4.4 | 8.3 |
| DREAMM-2 | 4.1 | 4.2 | 0.0 | 8.3 | 8.3 | 14.8 | 14.7 | 4.1 | 4.3 | 8.3 |
| Scenario 3.2: $(p_1, p_2) = (\mathbf{0.30}, 0.30)$ | | | | | | | | | | |
| TODO | **64.3** | 22.4 | 8.5 | 27.2 | 30.6 | 19.5 | 19.7 | 89.4 | 90.8 | 95.2 |
| TODO-BB | **59.3** | 26.0 | 8.2 | 32.5 | 35.7 | 19.3 | 19.3 | 74.5 | 75.0 | 93.5 |
| DREAMM-2 | **52.3** | 41.3 | 0.0 | 47.7 | 47.7 | 19.3 | 19.3 | 74.4 | 74.7 | 93.6 |
| Scenario 3.3: $(p_1, p_2) = (\mathbf{0.30}, 0.35)$ | | | | | | | | | | |
| TODO | **52.9** | 33.3 | 11.6 | 35.5 | 40.2 | 19.7 | 19.9 | 91.4 | 96.3 | 97.8 |
| TODO-BB | **49.4** | 36.1 | 11.3 | 39.3 | 43.8 | 19.3 | 19.7 | 74.5 | 87.2 | 96.8 |
| DREAMM-2 | **40.4** | 56.5 | 0.0 | 59.6 | 59.6 | 19.3 | 19.7 | 74.6 | 87.4 | 96.9 |
| Scenario 3.4: $(p_1, p_2) = (0.30, \mathbf{0.50})$ | | | | | | | | | | |
| TODO | 16.8 | **73.2** | 9.9 | 16.9 | 20.9 | 19.9 | 20.0 | 90.6 | 99.8 | 99.9 |
| TODO-BB | 17.9 | **70.0** | 11.8 | 18.2 | 22.9 | 19.3 | 20.0 | 74.5 | 99.2 | 99.7 |
| DREAMM-2 | 12.2 | **87.7** | 0.0 | 12.3 | 12.3 | 19.3 | 20.0 | 74.5 | 99.3 | 99.9 |
| Scenario 3.5: $(p_1, p_2) = (0.10, \mathbf{0.30})$ | | | | | | | | | | |
| TODO | 5.7 | **71.1** | 2.3 | 26.6 | 27.5 | 16.8 | 19.4 | 20.0 | 78.4 | 79.1 |
| TODO-BB | 2.5 | **72.9** | 0.8 | 26.4 | 26.7 | 14.7 | 19.3 | 4.1 | 75.0 | 76.1 |
| DREAMM-2 | 2.0 | **74.1** | 0.0 | 25.9 | 25.9 | 14.7 | 19.3 | 3.9 | 75.0 | 76.1 |
| Scenario 3.6: $(p_1, p_2) = (0.30, \mathbf{0.60})$ | | | | | | | | | | |
| TODO | 4.6 | **90.5** | 4.8 | 4.6 | 6.6 | 19.9 | 20.0 | 89.6 | 100.0 | 100.0 |
| TODO-BB | 6.3 | **87.5** | 6.2 | 6.3 | 8.8 | 19.3 | 20.0 | 74.5 | 99.9 | 100.0 |
| DREAMM-2 | 3.7 | **96.3** | 0.0 | 3.7 | 3.7 | 19.4 | 20.0 | 74.8 | 100.0 | 100.0 |
| Scenario 3.7: $(p_1, p_2) = (\mathbf{0.30}, 0.33)$ | | | | | | | | | | |
| TODO | **57.8** | 28.7 | 10.4 | 31.8 | 36.0 | 19.7 | 19.8 | 90.7 | 94.5 | 96.9 |
| TODO-BB | **53.4** | 31.6 | 10.6 | 36.1 | 40.3 | 19.3 | 19.6 | 74.5 | 82.9 | 95.5 |
| DREAMM-2 | **45.5** | 50.2 | 0.0 | 54.5 | 54.5 | 19.3 | 19.6 | 74.2 | 82.5 | 95.7 |
| Scenario 3.8: $(p_1, p_2) = (\mathbf{0.35}, 0.30)$ | | | | | | | | | | |
| TODO | **76.0** | 14.0 | 7.0 | 17.0 | 19.8 | 19.7 | 19.8 | 94.6 | 92.1 | 96.9 |
| TODO-BB | **72.9** | 16.1 | 7.5 | 19.7 | 22.6 | 19.6 | 19.3 | 86.5 | 75.0 | 96.4 |
| DREAMM-2 | **66.9** | 29.9 | 0.0 | 33.1 | 33.1 | 19.7 | 19.3 | 87.2 | 74.3 | 96.9 |
| Scenario 3.9: $(p_1, p_2) = (\mathbf{0.50}, 0.30)$ | | | | | | | | | | |
| TODO | **93.9** | 2.3 | 2.3 | 3.8 | 4.8 | 19.9 | 19.9 | 98.4 | 90.9 | 98.5 |
| TODO-BB | **95.0** | 2.3 | 2.5 | 2.5 | 3.5 | 20.0 | 19.3 | 99.2 | 75.0 | 99.8 |
| DREAMM-2 | **92.7** | 7.0 | 0.0 | 7.3 | 7.3 | 20.0 | 19.4 | 99.2 | 75.1 | 99.8 |

Table S8: Simulation results for the two-dose trials with $p_0 = 0.3$ and $p_1 = 0.5$. Optimal doses and correct selection decisions are highlighted in boldface. Selection %: Percentage of selecting the dose in the final analysis. SIR: Percentage of having an inconclusive result. IDR: Rate of incorrect decisions. WL: Weighted loss (with $w_l = 0.40$). ASS: Average sample size. Go %: Percentage of making a "go" decision in the futility monitoring, i.e., the per-dose power. FWER/OMP %: Percentage of trials identifying at least one dose as effective in the futility monitoring, which represents the family-wise type I error rate (FWER) in Scenario 4.1 and the overall monitoring power (OMP) in the other scenarios.

| Method | Selection % | | SIR | IDR | WL | ASS | | Go % | | FWER/OMP % |
|---|---|---|---|---|---|---|---|---|---|---|
| | $d_1$ | $d_2$ | | | | $d_1$ | $d_2$ | $d_1$ | $d_2$ | |
| Scenario 4.1: $(p_1, p_2) = (0.30, 0.30)$ | | | | | | | | | | |
| TODO | 4.5 | 5.4 | 0.1 | 9.9 | 9.9 | 20.2 | 23.2 | 4.6 | 6.4 | 9.9 |
| TODO-BB | 2.3 | 2.6 | 0.0 | 5.0 | 5.0 | 23.4 | 23.5 | 2.4 | 2.6 | 5.0 |
| DREAMM-2 | 2.4 | 2.5 | 0.0 | 4.9 | 4.9 | 23.4 | 23.6 | 2.4 | 2.6 | 4.9 |
| Scenario 4.2: $(p_1, p_2) = (\mathbf{0.50}, 0.50)$ | | | | | | | | | | |
| TODO | **67.4** | 18.0 | 9.9 | 22.7 | 26.7 | 30.3 | 30.6 | 85.4 | 86.9 | 95.3 |
| TODO-BB | **52.4** | 27.5 | 6.9 | 40.7 | 43.5 | 30.1 | 30.1 | 62.9 | 63.7 | 86.7 |
| DREAMM-2 | **47.0** | 39.4 | 0.0 | 53.0 | 53.0 | 30.1 | 30.2 | 63.8 | 63.2 | 86.5 |
| Scenario 4.3: $(p_1, p_2) = (\mathbf{0.50}, 0.55)$ | | | | | | | | | | |
| TODO | **56.3** | 27.6 | 14.0 | 29.6 | 35.2 | 30.6 | 30.8 | 86.9 | 94.9 | 98.0 |
| TODO-BB | **43.4** | 39.1 | 10.3 | 46.3 | 50.4 | 30.1 | 30.6 | 62.9 | 81.0 | 92.8 |
| DREAMM-2 | **35.5** | 58.2 | 0.0 | 64.5 | 64.5 | 30.1 | 30.6 | 63.3 | 82.3 | 93.6 |
| Scenario 4.4: $(p_1, p_2) = (0.50, \mathbf{0.70})$ | | | | | | | | | | |
| TODO | 15.9 | **73.0** | 11.1 | 16.0 | 20.4 | 30.8 | 31.0 | 86.3 | 99.9 | 100.0 |
| TODO-BB | 11.3 | **78.5** | 10.1 | 11.4 | 15.5 | 30.1 | 31.0 | 62.9 | 99.6 | 99.9 |
| DREAMM-2 | 6.3 | **93.6** | 0.0 | 6.4 | 6.4 | 30.2 | 31.0 | 63.3 | 99.6 | 99.9 |
| Scenario 4.5: $(p_1, p_2) = (0.30, \mathbf{0.50})$ | | | | | | | | | | |
| TODO | 6.0 | **68.7** | 2.4 | 28.9 | 29.8 | 25.8 | 30.0 | 11.8 | 76.2 | 77.1 |
| TODO-BB | 1.7 | **62.4** | 0.4 | 37.1 | 37.3 | 23.4 | 30.1 | 2.4 | 63.7 | 64.5 |
| DREAMM-2 | 1.4 | **63.7** | 0.0 | 36.3 | 36.3 | 23.4 | 30.2 | 2.5 | 64.1 | 65.0 |
| Scenario 4.6: $(p_1, p_2) = (0.50, \mathbf{0.80})$ | | | | | | | | | | |
| TODO | 2.7 | **94.4** | 2.9 | 2.7 | 3.9 | 30.7 | 31.0 | 85.4 | 100.0 | 100.0 |
| TODO-BB | 1.7 | **95.5** | 2.8 | 1.7 | 2.8 | 30.1 | 31.0 | 62.9 | 100.0 | 100.0 |
| DREAMM-2 | 0.9 | **99.1** | 0.0 | 0.9 | 0.9 | 30.2 | 31.0 | 63.7 | 100.0 | 100.0 |
| Scenario 4.7: $(p_1, p_2) = (\mathbf{0.50}, 0.53)$ | | | | | | | | | | |
| TODO | **61.4** | 23.1 | 12.5 | 26.1 | 31.1 | 30.5 | 30.7 | 86.1 | 92.3 | 97.0 |
| TODO-BB | **47.4** | 34.2 | 8.7 | 43.9 | 47.4 | 30.1 | 30.5 | 62.9 | 74.3 | 90.3 |
| DREAMM-2 | **41.2** | 50.1 | 0.0 | 58.8 | 58.8 | 30.2 | 30.5 | 64.2 | 75.1 | 91.3 |
| Scenario 4.8: $(p_1, p_2) = (\mathbf{0.55}, 0.50)$ | | | | | | | | | | |
| TODO | **82.0** | 8.4 | 6.8 | 11.2 | 13.9 | 30.6 | 30.7 | 93.9 | 87.7 | 97.2 |
| TODO-BB | **71.3** | 15.0 | 6.9 | 21.8 | 24.6 | 30.6 | 30.1 | 81.5 | 63.7 | 93.2 |
| DREAMM-2 | **65.4** | 27.6 | 0.0 | 34.6 | 34.6 | 30.7 | 30.1 | 81.3 | 63.1 | 93.0 |
| Scenario 4.9: $(p_1, p_2) = (\mathbf{0.70}, 0.50)$ | | | | | | | | | | |
| TODO | **97.6** | 0.4 | 0.6 | 1.9 | 2.1 | 30.7 | 30.7 | 98.5 | 86.3 | 98.5 |
| TODO-BB | **97.6** | 0.9 | 1.3 | 1.1 | 1.6 | 31.0 | 30.1 | 99.5 | 63.7 | 99.8 |
| DREAMM-2 | **95.9** | 4.0 | 0.0 | 4.1 | 4.1 | 31.0 | 30.2 | 99.7 | 64.4 | 99.9 |

Table S9: Possible trial outcomes for comparing three active doses.

| Unconstrained scenario | | | Monotone scenario | | |
|---|---|---|---|---|---|
| $d_1$ versus $d_3$ | $d_2$ versus $d_3$ | Final selection | $d_1$ versus $d_3$ | $d_2$ versus $d_3$ | Final selection |
| Inferior | Inferior | $d_3$ | Inferior | Inferior | $d_3$ |
| Inferior | Non-inferior | $d_2$ | Inferior | Non-inferior | $d_2$ |
| Non-Inferior | Non-inferior | $d_1$ | Non-Inferior | Non-inferior | $d_1$ |
| Inferior | Inconclusive | Inconclusive | Inferior | Inconclusive | Inconclusive |
| Inconclusive | Non-inferior | Inconclusive | Inconclusive | Non-inferior | Inconclusive |
| Inconclusive | Inconclusive | Inconclusive | Inconclusive | Inconclusive | Inconclusive |
| Inconclusive | Inferior | ? | | | |
| Non-inferior | Inferior | ? | | | |
| Non-inferior | Inconclusive | ? | | | |

Table S10: Simulation results for the three-dose trials, with optimal doses and correct selection decisions highlighted in boldface. Selection %: Percentage of selecting the dose in the final analysis. SIR: Percentage of having an inconclusive result. IDR: Rate of incorrect decisions. WL: Weighted loss (with $w_l = 0.60$). ASS: Average sample size. Go %: Percentage of making a "go" decision in the futility monitoring, i.e., the per-dose power. FWER/OMP %: Percentage of trials identifying at least one dose as effective in the futility monitoring, which represents the family-wise type I error rate (FWER) in Scenario 5.1 and the overall monitoring power (OMP) in the other scenarios.

| Method | Selection % | | | SIR | IDR | WL | ASS | | | Go % | | | FWER/OMP % |
|---|---|---|---|---|---|---|---|---|---|---|---|---|---|
| | $d_1$ | $d_2$ | $d_3$ | | | | $d_1$ | $d_2$ | $d_3$ | $d_1$ | $d_2$ | $d_3$ | |
| Scenario 3.1: $(p_1, p_2, p_3) = (0.20, 0.20, 0.20)$ | | | | | | | | | | | | | |
| TODO | 1.3 | 2.3 | 6.2 | 0.2 | 10.0 | 10.0 | 14.7 | 16.1 | 17.3 | 1.4 | 3.9 | 10.0 | 10.0 |
| TODO-BB | 0.1 | 0.7 | 5.2 | 0.0 | 6.0 | 6.0 | 14.8 | 17.2 | 19.2 | 0.1 | 0.9 | 6.0 | 6.0 |
| Scenario 3.2: $(p_1, p_2, p_3) = (\mathbf{0.40}, 0.40, 0.40)$ | | | | | | | | | | | | | |
| TODO | **57.7** | 12.8 | 13.5 | 11.0 | 31.3 | 37.9 | 21.4 | 21.8 | 21.9 | 83.7 | 91.7 | 95.0 | 95.0 |
| TODO-BB | **40.5** | 15.3 | 22.0 | 7.5 | 51.9 | 56.4 | 21.3 | 21.9 | 22.0 | 53.9 | 71.1 | 85.3 | 85.3 |
| Scenario 3.3: $(p_1, p_2, p_3) = (\mathbf{0.40}, 0.45, 0.45)$ | | | | | | | | | | | | | |
| TODO | **49.6** | 19.4 | 15.5 | 14.0 | 36.5 | 44.8 | 21.4 | 21.9 | 22.0 | 88.1 | 97.0 | 98.5 | 98.5 |
| TODO-BB | **41.1** | 21.9 | 20.3 | 10.9 | 47.9 | 54.5 | 21.3 | 21.9 | 22.0 | 63.1 | 86.2 | 94.3 | 94.3 |
| Scenario 3.4: $(p_1, p_2, p_3) = (\mathbf{0.40}, 0.40, 0.45)$ | | | | | | | | | | | | | |
| TODO | **47.8** | 13.6 | 23.1 | 13.5 | 38.7 | 46.8 | 21.4 | 21.8 | 22.0 | 86.0 | 94.3 | 98.0 | 98.0 |
| TODO-BB | **35.4** | 15.6 | 32.0 | 10.1 | 54.4 | 60.5 | 21.3 | 21.9 | 22.0 | 56.9 | 76.1 | 93.2 | 93.2 |
| Scenario 3.5: $(p_1, p_2, p_3) = (0.40, 0.40, \mathbf{0.60})$ | | | | | | | | | | | | | |
| TODO | 14.0 | 9.9 | **63.3** | 12.7 | 24.0 | 31.6 | 21.4 | 21.9 | 22.0 | 86.8 | 95.2 | 99.9 | 99.9 |
| TODO-BB | 11.6 | 10.4 | **67.2** | 10.8 | 22.1 | 28.5 | 21.3 | 21.9 | 22.0 | 59.0 | 79.7 | 99.8 | 99.8 |
| Scenario 3.6: $(p_1, p_2, p_3) = (0.40, \mathbf{0.60}, 0.60)$ | | | | | | | | | | | | | |
| TODO | 16.9 | **48.6** | 19.9 | 14.7 | 36.7 | 45.5 | 21.5 | 22.0 | 22.0 | 89.3 | 100.0 | 100.0 | 100.0 |
| TODO-BB | 19.2 | **50.3** | 15.9 | 14.4 | 35.2 | 43.9 | 21.4 | 22.0 | 22.0 | 70.1 | 99.6 | 99.9 | 99.9 |
| Scenario 3.7: $(p_1, p_2, p_3) = (0.20, \mathbf{0.45}, 0.45)$ | | | | | | | | | | | | | |
| TODO | 6.2 | **57.2** | 23.1 | 10.6 | 32.2 | 38.6 | 17.3 | 21.7 | 21.9 | 22.6 | 91.4 | 97.2 | 97.2 |
| TODO-BB | 2.4 | **58.3** | 23.8 | 7.6 | 34.1 | 38.7 | 17.3 | 21.8 | 22.0 | 5.0 | 80.0 | 92.0 | 92.0 |
| Scenario 3.8: $(p_1, p_2, p_3) = (0.15, \mathbf{0.45}, 0.45)$ | | | | | | | | | | | | | |
| TODO | 1.8 | **62.1** | 23.7 | 9.1 | 28.9 | 34.3 | 15.3 | 21.6 | 21.9 | 8.4 | 89.9 | 96.7 | 96.7 |
| TODO-BB | 0.5 | **60.5** | 24.4 | 6.1 | 33.4 | 37.0 | 15.3 | 21.7 | 22.0 | 1.0 | 78.5 | 91.5 | 91.5 |
| Scenario 3.9: $(p_1, p_2, p_3) = (0.40, 0.40, \mathbf{0.70})$ | | | | | | | | | | | | | |
| TODO | 2.9 | 4.1 | **87.0** | 6.0 | 7.0 | 10.6 | 21.4 | 21.9 | 22.0 | 86.2 | 94.7 | 100.0 | 100.0 |
| TODO-BB | 2.8 | 4.8 | **86.9** | 5.5 | 7.5 | 10.9 | 21.3 | 21.9 | 22.0 | 59.1 | 79.8 | 100.0 | 100.0 |

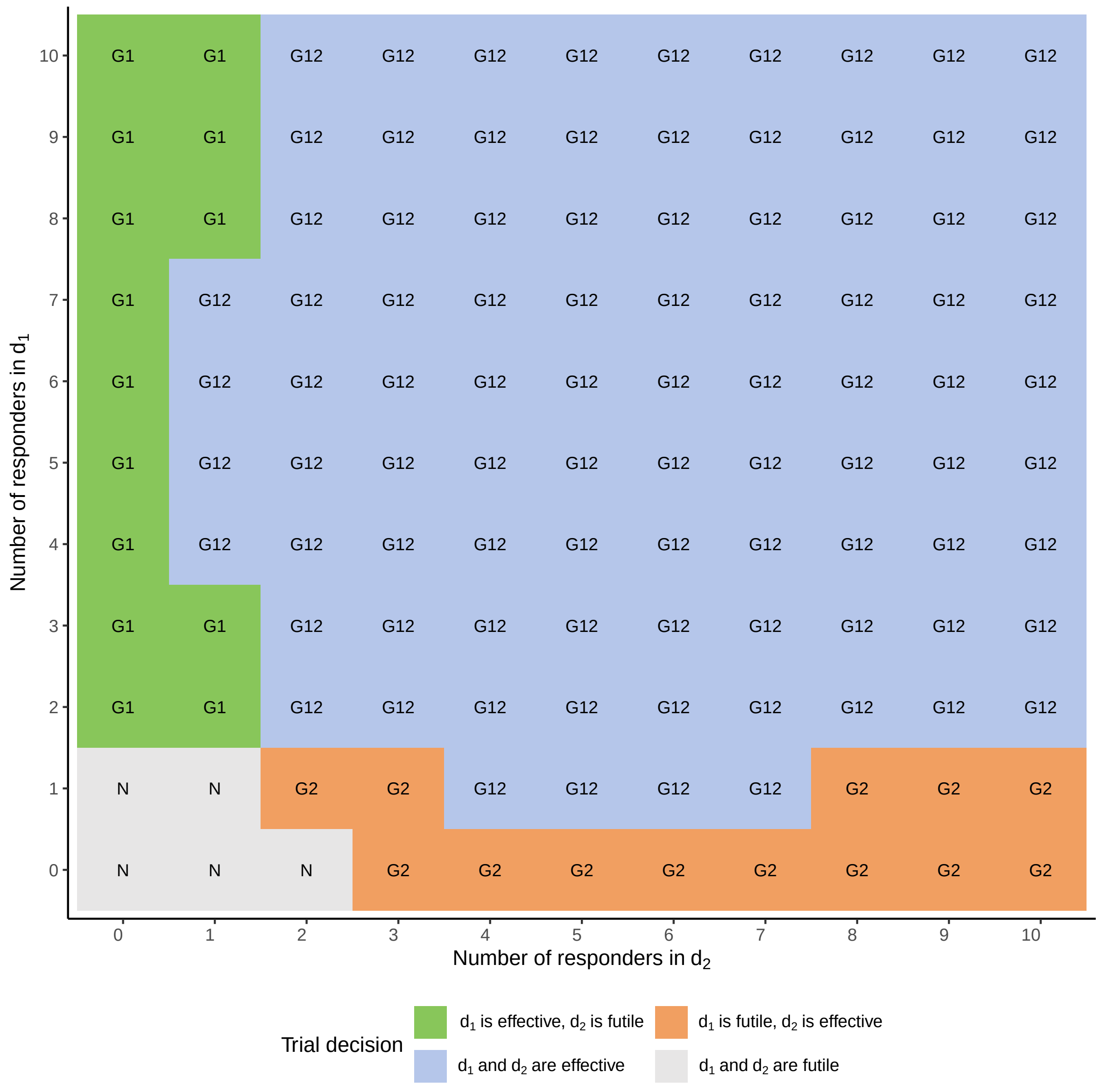


Figure S1: Interim analysis decision plot based on the number of efficacy responders in the proposed design for the two-dose trial described in Section 5.1. Here, the text $G1$ indicates "Go with $d_1$", $G2$ indicates "Go with $d_2$", and $G12$ indicates "Go with both $d_1$ and $d_2$".

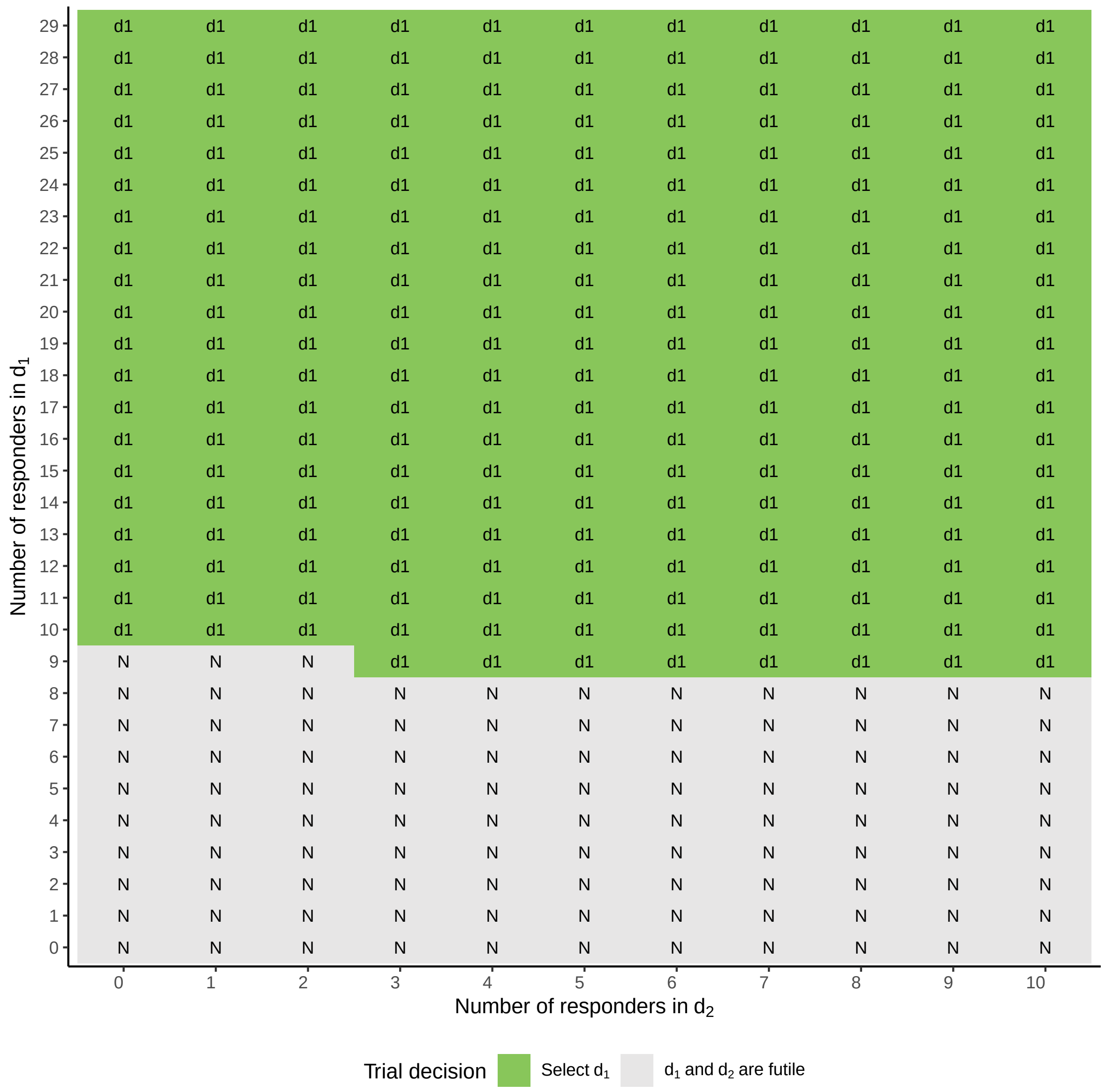


Figure S2: Final analysis decision plot based on the number of efficacy responders in the proposed design, assuming only dose $d_1$ passes the per-dose monitoring during the interim analysis, for the two-dose trial described in Section 5.1.

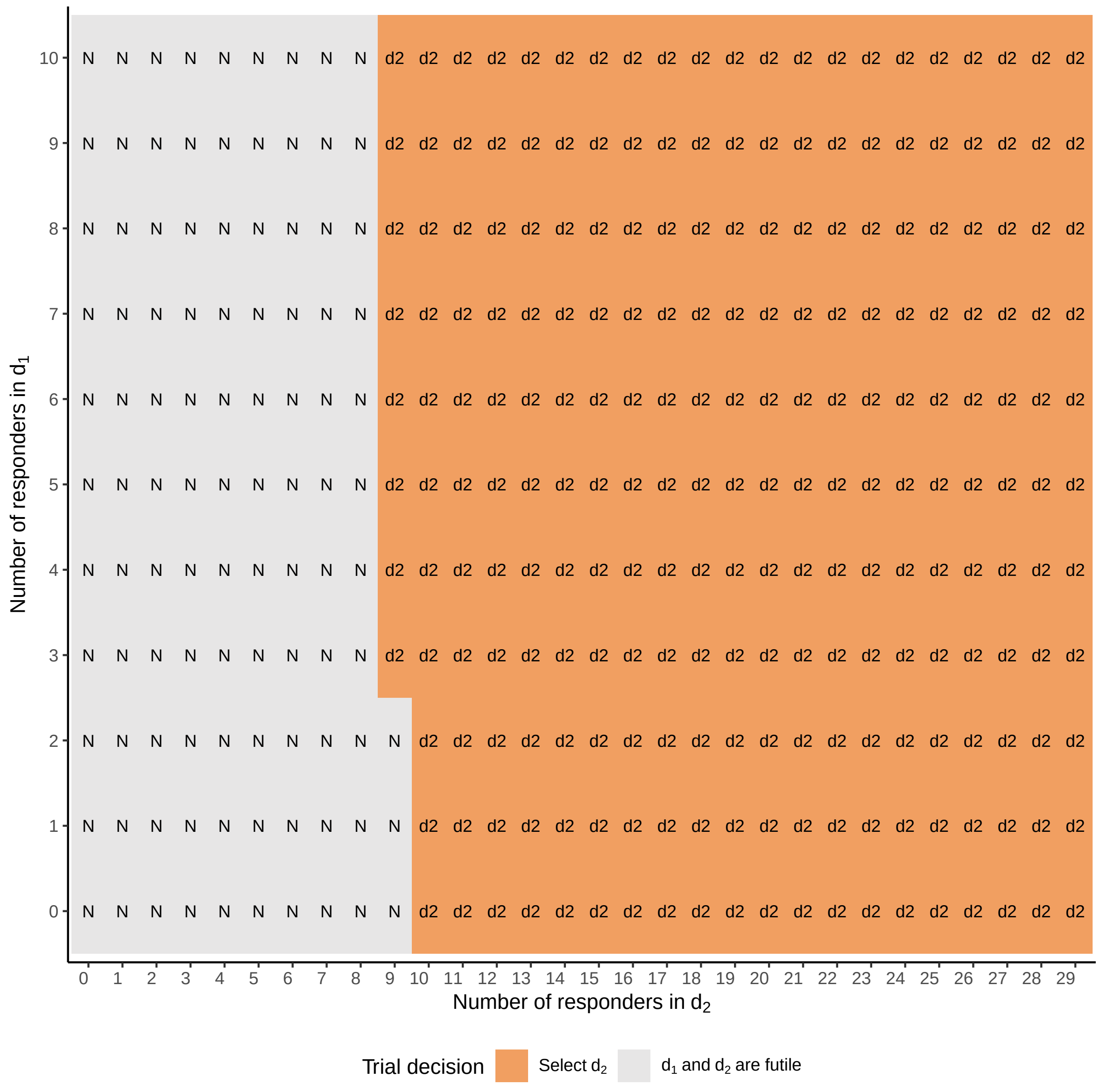


Figure S3: Final analysis decision plot based on the number of efficacy responders in the proposed design, assuming only dose $d_2$ passes the per-dose monitoring during the interim analysis, for the two-dose trial described in Section 5.1.

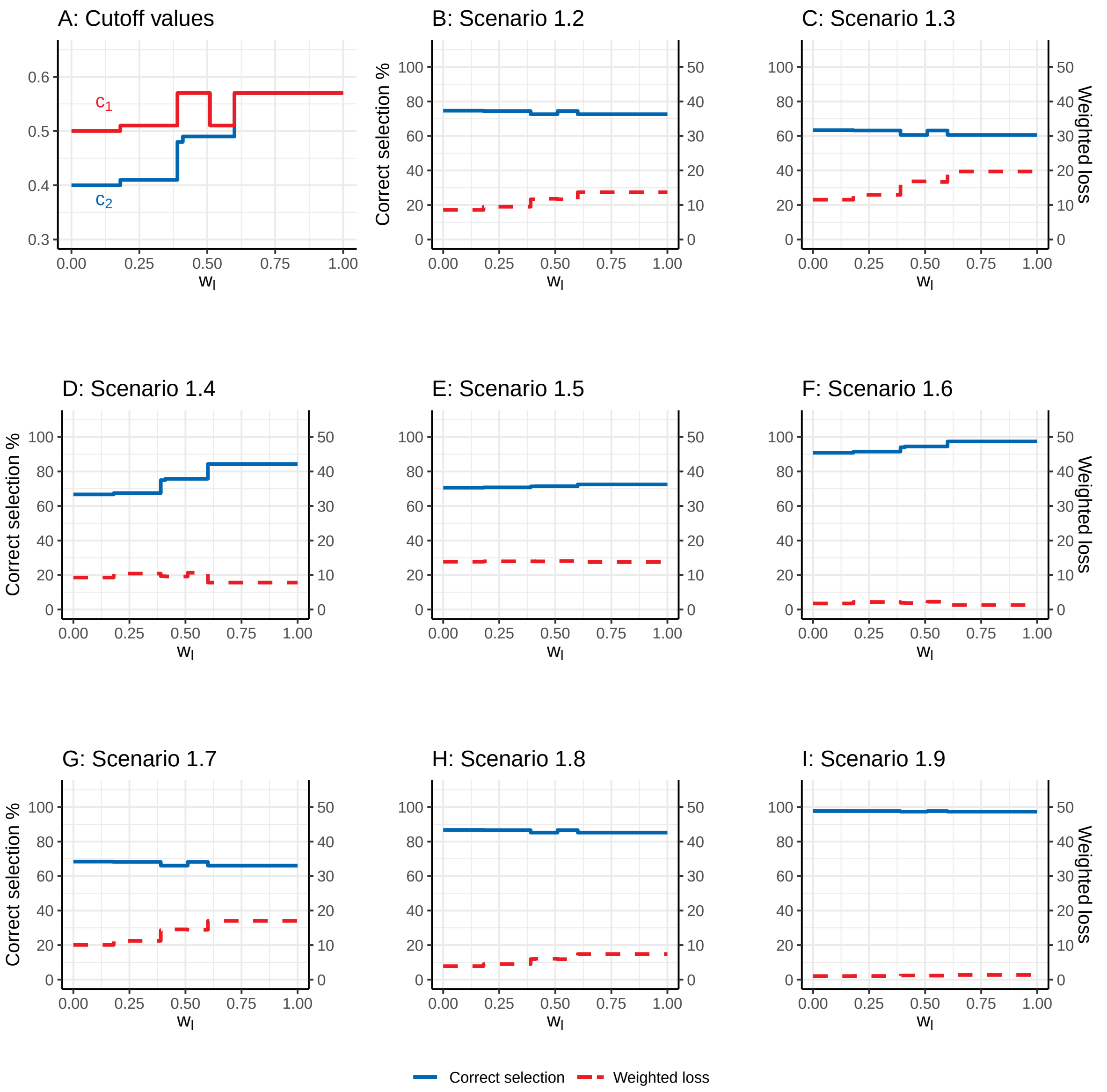


Figure S4: The influence of $w_l$ on the cutoff values and the selection percentage of the optimal dose with a sample size of 36.

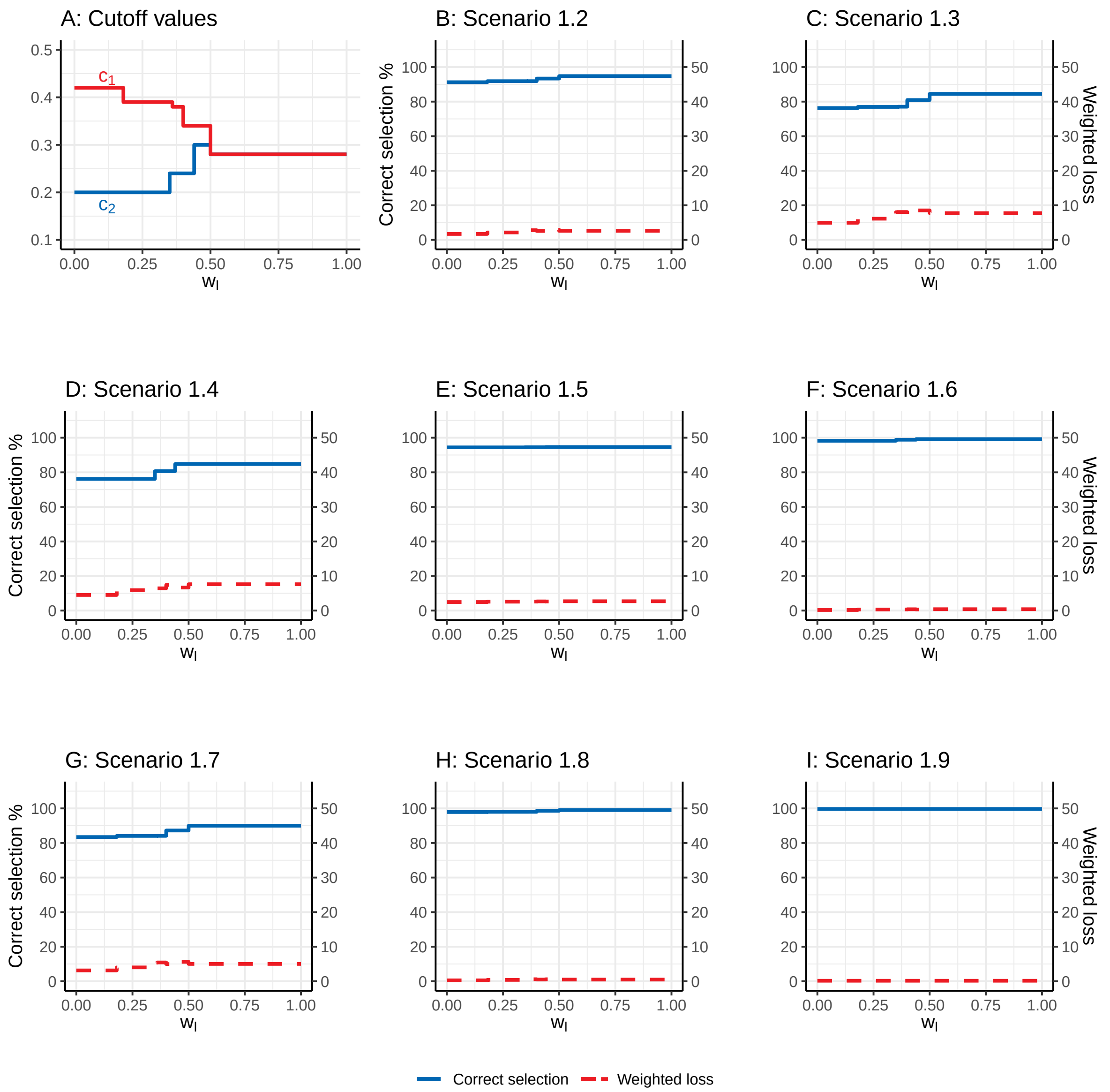


Figure S5: The influence of $w_l$ on the cutoff values and the selection percentage of the optimal dose with a sample size of 91.

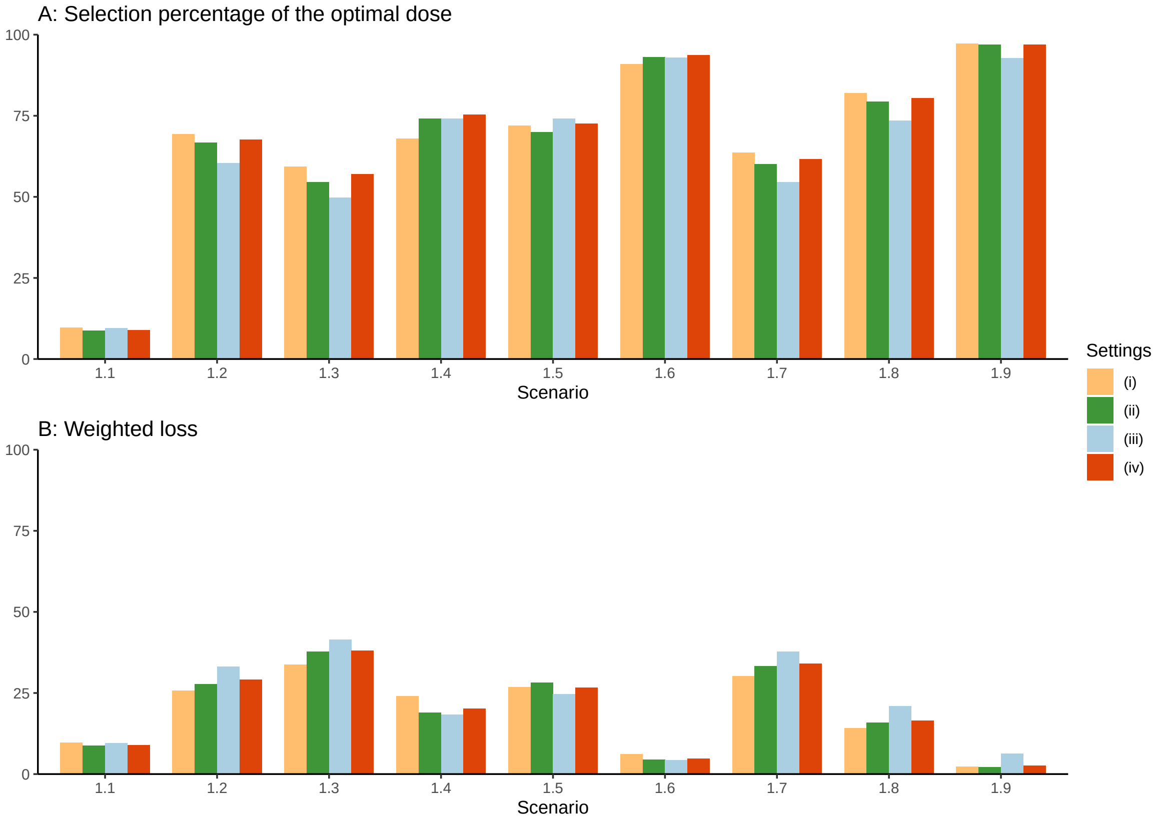


Figure S6: Results of sensitivity analysis for Scenarios 1.1 to 1.9 with a sample size of 28. $(i)$ $\theta = \Phi^{-1}(0.4)$. $(ii)$ $\tau^2 = 0.5$. $(iii)$ $\tau^2 = 5$. $(iv)$ Main result.

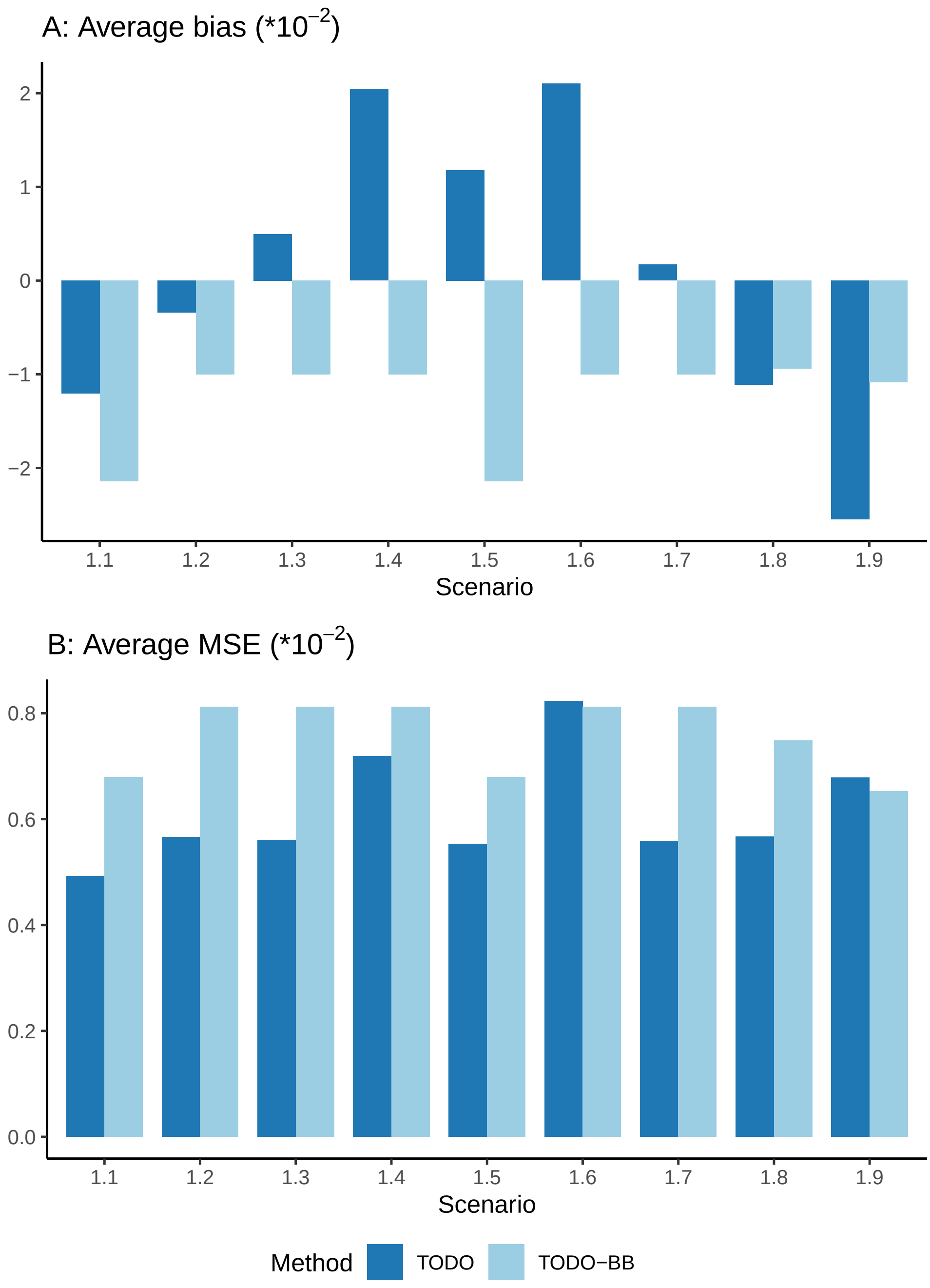


Figure S7: The average bias and MSE for Scenarios 1.1 to 1.9 with a sample size of 35.

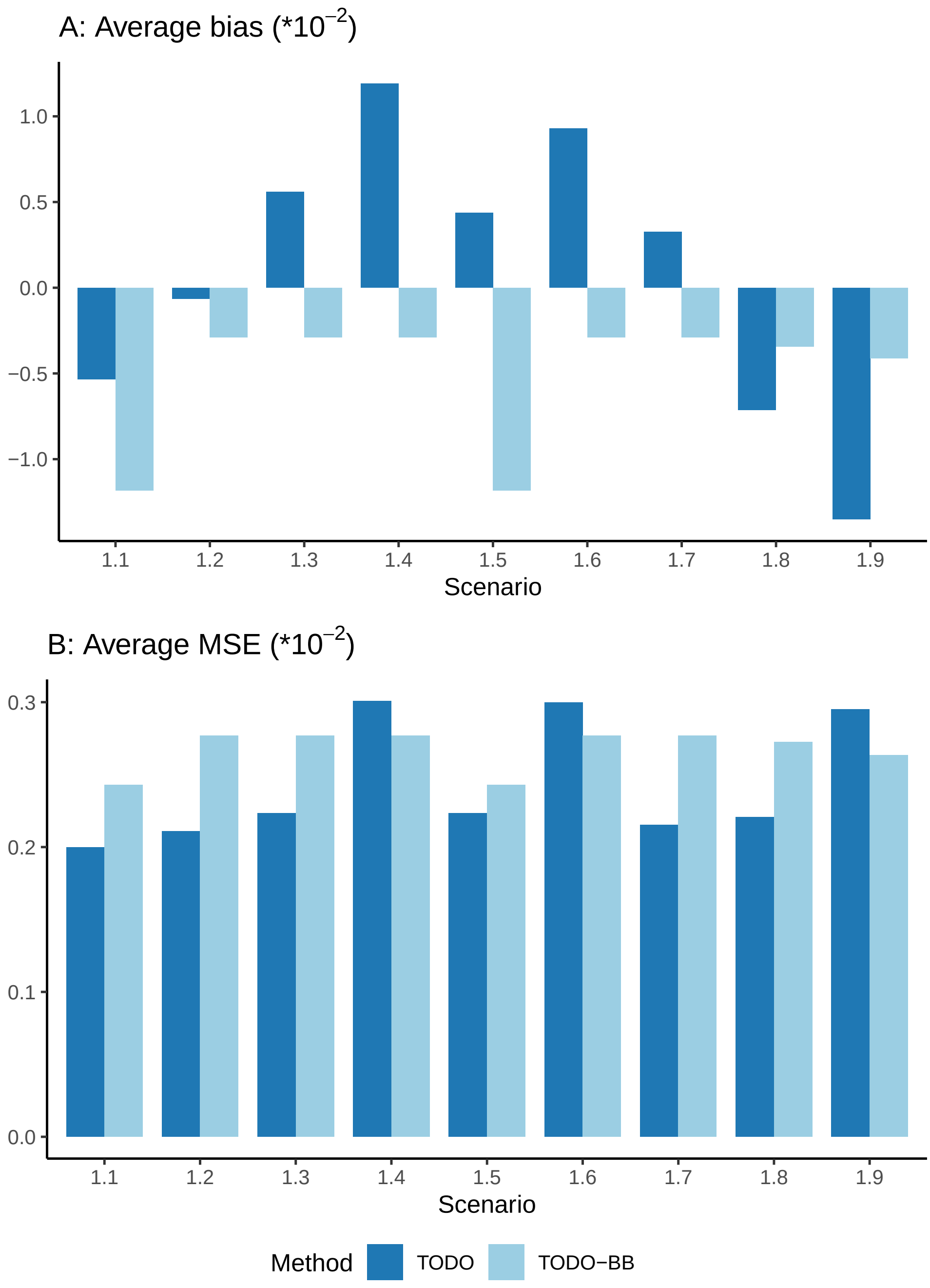


Figure S8: The average bias and MSE for Scenarios 1.1 to 1.9 with a sample size of 91.

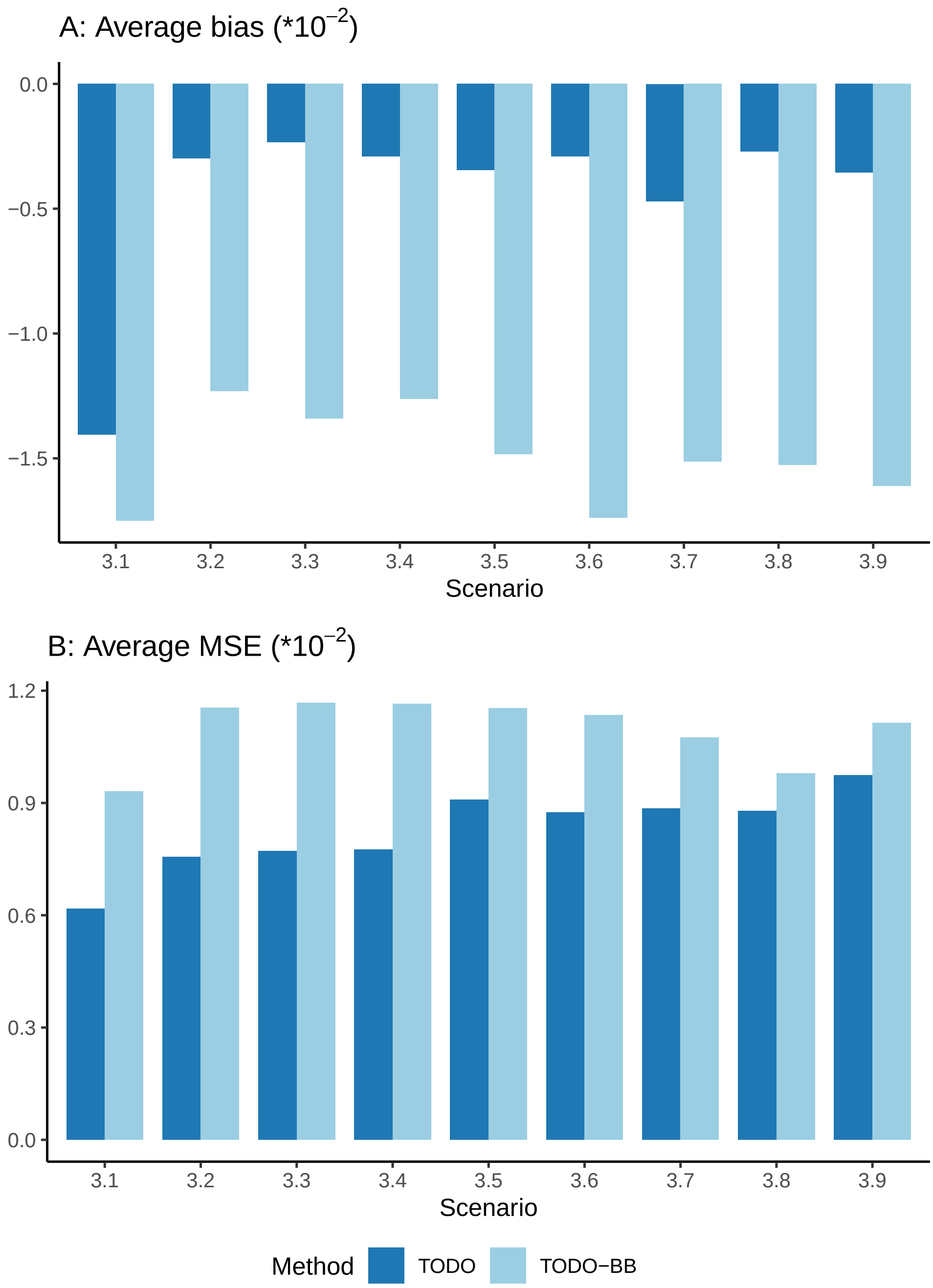


Figure S9: The average bias and MSE for Scenarios 5.1 to 5.9 with a sample size of 22.

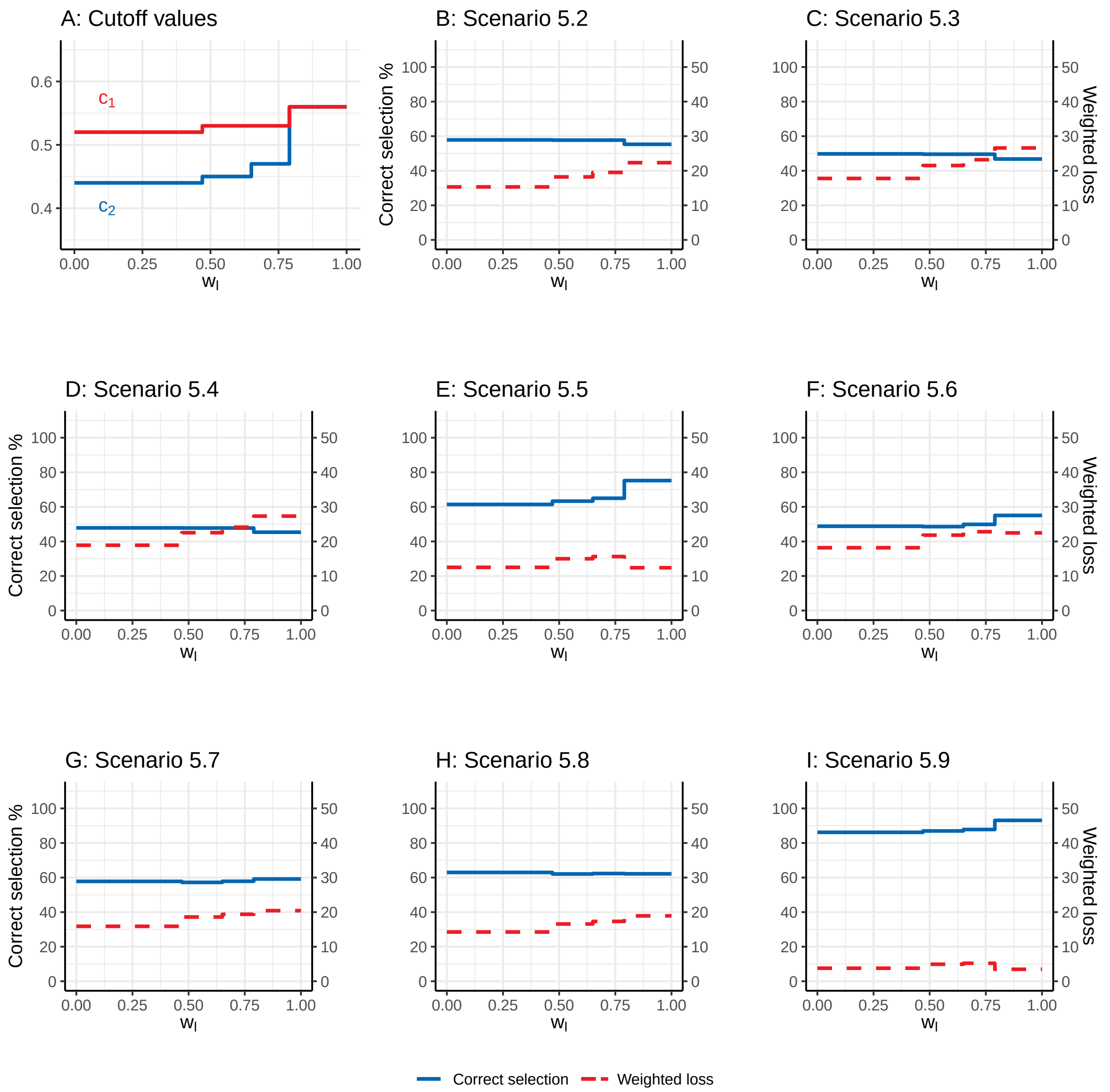


Figure S10: The influence of $w_l$ on the cutoff values and the selection percentage of the optimal dose for Scenarios 5.1 to 5.9 with a sample size of 22.
